
JOURNAL INFORMATION
==============================
NLM Title Abbreviation: JBRA Assist Reprod
Journal ID: JBRA Assist Reprod
NLM Journal ID: 101684552
Journal ID: jbra

JBRA Assisted Reproduction

ISSN: 1517-5693
EISSN: 1518-0557
Publisher: Brazilian Society of Assisted Reproduction

ARTICLE INFORMATION
==============================
PMCID: PMC5473709
PMID: 28609283
DOI: 10.5935/1518-0557.20170031
Article version: 1
Subjects: Poster Presentations

Poster Presentations: Abstracts of the 13th Red Lara Taller General, Buenos Aires, Argentina, 26-28 April 2017

Publication date: 2017 Apr-Jun
Print publication date: 2017 Apr-Jun
Volume: 21
Issue: 2
First page: 142
Last page: 160
License: This is an Open Access article distributed under the terms of the Creative Commons Attribution License, which permits unrestricted use, distribution, and reproduction in any medium, provided the original work is properly cited.
License URL: https://creativecommons.org/licenses/by/4.0/

==============================


P-01. Single step versus sequential culture medium: what is the best choice to improve ongoing pregnancy rate? A systematic review and meta-analysis

Dieamant F. 1 2
Petersen C.G. 1 2
Mauri A.L. 1 2
Conmar V. 1
Matilla M. 1
Vagnini L.D. 2
Renzi A. 2
Petersen B. 2
Ricci J. 1
Oliveira J.B.A. 1 2
Baruffi R.L.R. 1 2
Franco Jr. J. G. 1 2
1 Centre for Human Reproduction Prof Franco Jr, Research, Ribeirao Preto, Brazil
2 Paulista Center for Diagnosis Research and Training, Research, Ribeirao Preto, Brazil

P-02. Vaginismus in Assisted Reproductive Technologies Centers: an invisible population to be cared

Souza M.C.B. de 1
Gusmão M.C.G. 1
Antunes R.A. 1
Souza M.M. de 1
Rito A.L.S. 1
Lira P. 1
Mancebo A.C.A. 1
Tamm M.A. 1
Panaino T.R. 1
Bahia M.J. 1
1 Fertipraxis Reprodução Humana, Rio de Janeiro, Brazil

P-03. Application of platelet-rich plasma as coordinating in the endometrial preparation of patients with refractory endometrium

Molina A. 1 2
Sánchez J. 1 2
Sánchez W. 1 2
Vielma V. 1 2
1 Unidad de Asistencia Materno Reproductiva (UNAMATER) Mérida. Venezuela
2 Instituto Venezolano de Fertilidad Caracas (IVF CARACAS) Caracas D.C. Venezuela

P-04. How the general semen quality influence the blastocyst formation rate: analysis of 4205 IVF cycles

Piccolomini M. 1
Faneli M. 1
Bonetti T. 1 2
Motta E. 1 2
Serafini P. 1 3
Alegretti J.R. 1
1 Huntington - Medicina Reprodutiva. São Paulo, SP, Brazil
2 Disciplina de Ginecologia Endocrinológica, Departamento de Ginecologia, Escola Paulista de Medicina da Universidade Federal de São Paulo (UNIFESP-EPM), São Paulo, SP, Brazil
3 Disciplina de Ginecologia, Departamento de Obstetrícia e Ginecologia, Faculdade de Medicina, Universidade de São Paulo (FMUSP), São Paulo, SP, Brazil

P-05. Embryo development until blastocyst stage with and without renewal of single medium on day 3

Peña F. 1
Dávalos R. 1
Rechkemmer A. 1
Ascenzo A. 1
Gonzales M. 2
1 Laboratorio de Reproducción Asistida, Clínica Miraflores, Lima, Peru
2 Laboratorio de Biotecnología Animal, Facultad de Ciencias, Universidad Ricardo Palma, Lima, Peru

P-06. Development of a database as an electronic medical record for infertility patients and allowing progress notes and case report forms and reusing the data for clinical research

**Objective:** This study aimed to evaluate if single embryo culture medium (Single-ECM) better than sequential medium (Sequential-ECM), and can it improve ongoing pregnancy rates in patients undergoing assisted reproductive technique (ART) procedures.
**Methods:** A systematic review based on electronic searches of databases was conducted and four randomized controlled trails (RCTs) were identified as targets for data extraction and meta-analysis. The primary outcome was ongoing pregnancy rate. Secondary outcomes included clinical pregnancy and miscarriage rates. The outcomes for ongoing pregnancy rate are also demonstrated according with the moment of the embryo transfers were performed: cleavage stage (day 2/3) and/or blastocyst stage (day 5/6).
**Results:** General results demonstrated no significant differences between Single-ECM and Sequential-ECM groups for clinical pregnancy (RR=1.09; 95%CI=0.83 - 1.44; *P*=0.53), ongoing pregnancy (RR=1.11; 95%CI=0.87 – 1.40; *P*=0.39), and miscarriage rates (RR=0.89; 95%CI=0.44 – 1.81; *P*=0.74). Including only RCTs in which the embryo transfer was performed at the blastocyst stage, no significant difference was found for ongoing pregnancy rate (RR=1.29; 95%CI=0.93 – 1.78; *P*=0.12) between Single-ECM and Sequential-ECM groups.
**Conclusion:** The embryo culture medium employed, Single-ECM or Sequential-ECM, does not improve ongoing pregnancy rates in patients undergoing an ART cycle.

**Objective:** Genital and sexual pain is still neglected. Consequences can be dramatic since a direct relation between infertility and sexual dysfunction can be reciprocal. This is the first study focusing in identifying vaginismus cases in an ART scenario and intra-cycle interventions as a comprehensive, integrated and patient-centered perspective.
**Methods:** Observational prospective study. Among 425 initiated IVF/ICSI cycles and 226 frozen embryo transfers from January 1, 2015 to December 31, 2016, we identified 07 cases of vaginismus. In a 6 months' time a Survey Monkey questionnaire was twice sent to 228 Latin American ART centers. We were interested in knowing how often women with vaginismus were arriving at ART centers and what was the perception of their presence.
**Results:** The low percentage of questionnaires responses (24/10.5%) states as not relevant the questioning about difficulties in performing ultrasound controls or the need for sedated transfers. Although 81% agreed that these situations occur at low incidence, no references to vaginismus, dyspareunia or any sexual dysfunction were made. Our multi-professional team handled 07 cases of vaginismus in which all women had superior degree, average 37.8 years old and were married for at least 4 years. Although two of them related they could tolerate intercourse, all of them related previous treatments, such as vaginal dilators (3 of them), psychotherapy (4 of them) or psychiatric assistance (1 patient). Staff working was built to minimize their sufferings.
**Conclusion:** Gentle care and sensitive listening should be included in multi-professional teams to identify these women and to offer these couples a better quality treatment.

**Objective:** To improve endometrial quality and implantation rates after the application of platelet-rich plasma in patients with refractory endometrium.
**Methods:** 19 patients undergoing in vitro fertilization aged between 33 and 45 years with a history of refractory endometrium, to whom platelet rich plasma was given by infusion with a catheter into the uterine cavity on the tenth day of hormone replacement therapy, and then 72 hours after the first application.
**Results:** Endometrial thicknesses of >7mm were reported with the first application and in all cases, endometrial thicknesses of >9mm were evident after the second application. The total of the study group qualified for Embryo Transfer at the blastocyst stage. 73.7% of positive pregnancy tests of which 26.3% for live births, 26.3% for ongoing pregnancies, 10.5% for biochemical pregnancies, 5.3% for anembryonic pregnancy and 5.3% for fetal death (16 weeks).
**Conclusions:** The application of platelet rich plasma and its biostimulating effects on the endometrial microvasculature seems to offer encouraging benefits to refractory endometrium, providing an increase in endometrial receptivity and a consequent increase in implantation rates. As an autologous resource, that are easy to obtain and inexpensive, it is recommended to be included in the different protocols for endometrial preparation, including those in which a natural cycle is preferred.

**Objective:** To evaluate retrospectively the rate of cleavage embryo and blastocyst formation rate in different sperm quality groups, in a large cohort of oocytes.
**Methods:** Retrospective cohort study involving 4205 IVF cycles. Four groups were designed according to semen quality: normal, mild alteration, severe alteration and epididymis. All cycles intended to extended embryo culture until blastocyst stage and the embryo development was observed.
**Results:** Regards the cleavage rate, normal semen and mild alteration groups were equivalent, as the severe alteration and epididymis groups. When the blastocyst formation rate was evaluated, the rates were decreasing as the semen quality was declining. In normal semen group, 79.9% of cycles had at least one blastocyst formed, while the mild alteration (75.6%), severe alteration (76.4%) and epididymis (76.8%) groups presented significantly lower percentage of cycles with at least one blastocyst. Multivariate logistic regression showed that each cleaved embryo on D3 more, double the chance of having at least one blastocyst. Also, the chance of having at least one blastocyst was decreased when semen presented mild or severe alterations.
**Conclusion:** We suggest the general quality of sperm is a good predictor of blastocyst formation, significantly decrease the chance of having at least one blastocyst at the end of the cycle. Hence, we suggest the necessity of consider the general quality of semen and the number of cleaved embryos in the decision making process of cleaved embryos transfer or follow to extended culture to blastocyst transfer.

**Objective:** To evaluate the effect of embryonic development up to the blastocyst stage in a single culture medium without renewal on day 3
**Methods:** The study was carried out in the Assisted Reproduction Laboratory of Clínica Miraflores during the period from April to December 2016. A total of 589 human embryos obtained from 82 couples undergoing IVF / ICSI treatment with oocytes donated were used. All couples were randomly divided into two groups: group 1, with renewal and group 2, without renewal.
**Results:** Significant differences were not observed between pregnancy rates, implantation rates and blastocyst formation.
**Conclusion:** The results obtained in this study do not show statistically significant differences between both groups of cultures with respect to all parameters evaluated. However, we can observe a non-signifficant trend that favors the rate of clinic pregnancy and positive pregnancy (β-hCG) in the without renewal group.

**V. Chaquiriand[1]**

*[1]Centro de Esterilidad Montevideo, Uruguay.*

**Objective:** Since 2013 Uruguay has recognized infertility as a disease. Social security must cover diagnose and treatments in public and private institutions. The number of patients assisted for this problem increased as well as the number of cycles. In order to optimize our work as well as having a tool for making reports or retrieve data for medical research we developed since 2011 a database that we continue to modify making new versions, letting us ameliorate the use and the information recorded.
**Methods:** We used Filemaker Pro Advanced, last version 15, and we created many models in order to complete during consultation information about the couple (see picture). The objective was to standardize a complete clinical history and exam, and make easy to fill data. We put dosages, studies, and progress notes. And we made made models for stimulations, follicle counts, endometrial follow up, laboratory results and pregnancy results. It can be used in any language the professional could want to or the institution needs it. It's just a translating some of the items in order of having it in the wanted language.
**Results:** By now we have 2996 records of patients, their results and their evolution. We also have registered 1672 stimulation forms, and their results. Medical records for the moment are filled after consultation, because in every institution we work we have to fill the local medical file. So we have to do it after. It allows us to review the case, to search sometimes for information and to think about it again after patients leave. Once we have the indication of treatment we make the summary to send to the institution that will pay for the treatment. Most of the data are directly taken from the record, so we just have to review it and sometimes to add some conclusions. The stimulation forms are filled while we make the sonographic follow up, as well as the medication indicated. Once we finish the stimulation we can generate a PDF file and send it to the patients or the institution as a report with just a few clicks. Those files can be filled in computers, but also in iPad or iPhone. Normally we use the iPad in consultation and we back-up in computers and in hard drive disks. Those back-ups are made every day, and in real time in the Cloud.
**Conclusion:** This database became a useful tool, that can be modified anytime if necessary, to get it better, and to help data standardization in order to recover information. It became an important tool that let us work better, to optimize time excluding redundant work, make clinical research and analyze our results. In the future, we hope to obtain important information due to the good number of cases registered.

## P-07. Randomized comparison of two commercial culture media (Cook and Vitrolife) for embryo culture after IMSI

**C.G. Petersen[1,2], A.L. Mauri[1,2], L.D. Vagnini[2], A. Renzi[2], B. Petersen[2], M. Matilla[1], V. Comar[1], J. Ricci[1], F. Dieamant[1], J.B.A. Oliveira[1,2], R.L.R. Baruffi[1,2], J. G. Franco Jr[1,2]**

**Objective:** A variety of studies randomizing women/cycles or oocytes/embryos have been conducted to compare different culture media for culturing embryos up to cleavage or blastocyst stages showing controversial results. A recent systematic review suggested that data in literature are insufficient to conclude the best culture medium for embryo quality, pregnancy and implantation. The objective of this study was to evaluate if there is any difference between two commercial culture medium regarding clinical outcomes after IMSI cycles.
**Methods:** A total of 120 patients, ≤39 years, undergoing ART treatment submitted to IMSI program were divided prospectively and at random into two groups: Group I (Cook media) and Group II (Vitrolife media).
**Results:** Our data demonstrated that there is no difference using all the medium from Cook or all the medium from Vitrolife, for culturing embryos till day 2 in the bench incubator at low O2 concentration, in relation of fertilization, embryos quality, pregnancy and implantation rates.
**Conclusion:** Both culture media used, Cook medium and Vitrolife medium, for the IMSI procedure and for later embryo culture with transfer on the second day are equally effective and can be used depending on the ease and availability of acquisition.

## P-08. Early serum progesterone and prolactin analysis at day 9 of follicular uptake as a predictor of success in fresh ICSI cycles

**P.A.S. Pérez[1], A.P. Ceschin[1]**

**Objective:** In order to obtain pregnancy, several factors are analyzed before and during the cycle of in vitro fertilization. Progesterone supplementation for adequate luteal phase support is indicated despite the presence of multiple corpus luteum in IVF stimulation cycles due to blockage caused by hypothalamic agonists and antagonists. The dosage of progesterone and prolactin on day 09 after the follicular retrieval could function as a predictive marker of success in fertility treatments. Objective: To analyze plasma levels of progesterone and prolactin nine days after follicular uptake and to evaluate their correlation with pregnancy rates in in vitro fertilization cycles.
**Methods:** A retrospective study was performed using data from 238 patients submitted to intracytoplasmic sperm injection (ICSI) at a private infertility clinic from January 2013 to December 2015. Hormonal measurements were performed on day 09 after follicular uptake. Data were compared to assess the correlation between prolactin and progesterone dosages and pregnancy rates.
**Results:** The ICSI pregnancy rate was 40.8% (n=38). No statistically significant difference was observed when correlating the success of the procedure with the prolactin dosage ($P$=0.71). However, progesterone showed a significant difference ($P$=0.021). The cutoff point indicated by the ROC curve fit according to which gestations would be identified is 25.95 ng/ml progesterone. The sensitivity of this point is 61.9% and the specificity is 57.4%.
**Conclusion:** Progesterone dosage may be one of the indicators of gestation on day 09 after follicular uptake. Such data may assist physicians in monitoring and provides suitable early gestational care.

## P-09. Healthy birth following intracytoplasmic sperm injection from ejaculated sperm after bilateral testicular sperm extraction failure

**J.F. de Macedo[1], G. C. de Macedo[2], G. C. de Macedo[2], L.A. Campos[1,3], O.C. Baltatu[3]**

**Introduction:** Oligoasthenoteratozoospermia is associated with decrease in pregnancy rate. We report a successful pregnancy after assisted reproduction with use of ejaculated sperm after failed bilateral testicular sperm extraction (TESE).
**Case report:** Patient(s): Couple with male factor infertility with severe oligoasthenoteratozoospermia: sperm count of 300.000/ml, 3% of sperm motility, 1% of sperm progressive motility with seminal processing, and progressive motility recovery rate of 100.000/ml. Intervention(s): Intracytoplasmic morphologically selected sperm injection (IMSI) with freshly ejaculated sperm retrieved after general anesthesia with intravenous doses of propofol (2.5 mg/kg) used for bilateral TESE. Main outcome measure(s): Fertilization, pregnancy, and birth after IMSI with ejaculated spermatozoa followed by failed bilateral TESE. Result(s): Bilateral TESE was indicated after low cell sperm count (1x106) and failed IVF cycle due to embryo development halting on the third day of IVF.

After the patient recovered from the general anesthesia for TESE, since only sperm with amorphous heads could be harvested, ejaculated sperm was obtained. A total of 1x106 ejaculated sperm count was obtained, which was further prepared for IMSI. From five fertilized eggs, one reached blastocyst stage. After a single blastocyst transfer, pregnancy succeeded and progressed to term with a healthy child.
**Comments:** With ejaculated sperm, possibly activated by general anesthesia, successful fertilization was accomplished by IMSI after failed bilateral TESE.

## P-10. Study of the main reasons for rejection

**M. Casado[1,2], B. Martínez[1,2], E. Criado[1,2]**

[1]*FIV Marbella, Spain.*
[2]*Ovobank, Spain.*

**Objectives:** Quantify the number of egg donor candidates who are excluded throughout the different phases of the process and the reasons for rejection.
**Methods:** Data from 921 donors are presented. All of them went through different selection filters. The first is an informative visit. Donors who pass this filter will undergo gynaecological examination and then a psychological interview.
**Results:** A descriptive investigation has found that of 921 applicants, 357 are not suitable, of which 225 are rejected in the informative visit. Of the remaining 132 who go on to have a gynaecological examination only 69 will be eligible, and of these, 45 will be rejected for having some type of severe genetic alteration and 24 will be unsuitable for psychological reasons. Only 61.24% of women who want to donate are eligible.
**Conclusion:** The egg donors selection process is exhaustive, a BMI>30 being the main reason for exclusion, assuming 10.1%. Followed by personal history 9.3%. As for the genetic motive, the percentage of rejected donors is lower, it is 4.9%. Currently many more mutations and polymorphisms are seen in the DNA due to the advances in science in this field, it is for that reason, that the accomplishment of genetic matching is carried out more and more thus obtaining that some alterations in the DNA do not suppose a reason of exclusion. For this reason, performing of genetic matching is carried out more and more so that certain alterations in the DNA do not suppose a reason for exclusion.

## P-11. Uninterrupted embryo culture. Development of blastocysts using a continuous culture system without medium renewal

**M.F. Veiga[1], A.Rivadeneira[1], S. Giordana[1], F. Neuspiller[1]**

[1]*IVI Buenos Aires - Argentina.*

**Objective:** The objective of the present study is to evaluate the effect of uninterrupted culture of embryos up to blastocyst stage without time-lapse technology.
**Methods:** We make a retrospective, analytical and longitudinal study of cycles of in vitro fertilization (IVF) performed between the 23rd of July and the 7th of December 2016 at IVI-BA. Cycles performed with patients fresh oocytes were included in the analyses as well as those performed with donated oocytes.
Embryos were obtained from IVF or ICSI cycles after controlled ovarian stimulation. Fertilization was evaluated 16-18 hours post insemination. Individual embryos were cultivated to the blastocyst stage in drops of 50 µl of GGT medium (LifeGlobal®). In the control group the medium was renewed on the third day of development, while in the study group there was no medium renewal. Embryo quality was assessed according ASEBIR criteria.
**Results:** The blastocyst development rate (BR), as well as the proportion of viable embryos (VE) and good quality embryos at day 5 (GQ) were significantly higher in the study group compared to the control group (BR: 75% vs 69%, VE: 45% vs 34%, GQ: 26% vs 16% respectively) ($P$<0.05; Fisher's exact test).
**Conclusion:** The study shows that there is an advantage in the uninterrupted embryo culture, which is reflected in a greater number of blastocysts, viable and good quality embryos.

## P-12. Importance of the diagnostic hysteroscopy in an in vitro fertilization program: experience in our center

**M. Blum-Narváez[1], T. Puga-Torres[1], B. Blum-Pinto[1], I. Arce-Burgos[1], B. Espinoza-Constante[1]**

**Objective:** To establish the diagnostic accuracy between transvaginal ultrasound and diagnostic hysteroscopy in patients undergoing assisted reproductive treatment.
**Methods:** A retrospective study was performed from 106 patients who underwent transvaginal sonography and subsequent diagnostic hysteroscopy before starting in vitro fertilization treatment. The hysteroscopy was performed with a flexible hysteroscope under conscious sedation. The results of both procedures were evaluated by the Mc Nemar statistical test with $P$<0.05.
**Results:** Of the total number of patients evaluated, 91 did not present uterine pathologies with transvaginal sonography; however from this group of patients, hysteroscopy revealed significant pathology in 31 cases (table 1). Knowing that pathologies located in the uterine cavity (table 2) could affect the implantation process, all patients with these pathologies underwent surgical hysteroscopy before the assisted reproduction procedure, obtaining a pregnancy rate of 75%.

**Table 1.** Correlation of findings from the diagnostic methods.

| Diagnostic Method | | | | Number of Cases |
|---|---|---|---|---|
| Sonography | | Diagnostic hysteroscopy | | |
| No Pathology | With Pathology | No Pathology | With Pathology | |
| X | | x | | 60 |
| | x | | x | 9 |
| | x | x | | 6 |
| X | | | x | 31 |

**Table 2.** Pathologies identified with diagnostic hysteroscopy.

| Pathology | Number of Cases |
|---|---|
| Endometrial Polyps | 23 |
| Endocervical polyps | 8 |
| Uterine Cavity Fibroids | 4 |
| Structural Uterine Anomaly | 1 |
| Combined Pathologies | 4 |

**Conclusion:** The obtained results allow us to infer that diagnostic hysteroscopy prevails over other diagnostic procedures that determine conditions of the uterine cavity.

## P-13. ART using washing semen in serodiscordant couples

**V. Reig[1], L.A. Proença[1], S.W. Flach[1], D.T. Kvitko[1], A. Tagliani-Ribeiro[1], L. Okada[1], R. Azambuja[1], A. Petracco[1], M. Badalotti[1]**

**Objective:** Semen washing is a safer reproductive strategy that discordant couples can use to achieve pregnancy through assisted reproductive technologies (ART). The objective of this work is to evaluate the pregnancy rate and the viral contamination of sperm in men with Human Immunodeficiency Virus (HIV), Human T-lymphotropic virus (HTLV),Hepatite C (HCV) and B virus (HBV) infection that underwent intrauterine insemination (IUI) or in vitro fertilization (IVF) with intracytoplasmic sperm injection (ICSI) using washed semen.
**Methods:** We evaluated 29 male patients in 56 IUI procedures, and ICSI often with subsequent frozen embryo transfer (FET), who underwent ART between November 2011 and December 2016. Of these, 15 were HIV positive, 8 HBV positive, 3 HCV positive, 2 HTLV positive, and 1 was positive for HBV and HCV. The sperm was centrifuged on a two-phase density gradient, washed with HEPES medium, and afterwards one part was cryopreserved in 8 straws and the other part was tested to detect viral particles. Non-infected women were tested before and after each procedure, as were the neonates at birth.
**Results:** The pregnancy rate of patients undergoing ICSI was 41%, FET was 30.8%, and no patient undergoing IUI became pregnant. After virological analysis, no virus particles were detected in any sample of the washing semen. No partner was infected through the procedures and none of the 10 births was infected in this study.
**Conclusions:** Sperm washing is shown to be a safe and effective option for reducing the risk of transmission in serodiscordant or concordant couples who wish to have a child.

## P-14. Effect of MACs on sperm DNA fragmentation and survival rate

**M.E. Grela[1], M.T. Olivieri[2], A. Díaz-Acacio[2], S. Bronfenmajer[2]**

**Objective:** To analyze the influence of MACS on the sperm DNA fragmentation and the survival rate, after sperm capacitation by discontinuous gradient centrifugation.
**Methods:** Twenty-two non-oligospermic semen samples were analyzed and sperm capacitation was performed, using discontinuous gradient centrifugation (DGC). After centrifugation, half of the sample was processed using the MiniMACS Starting Kit. Sperm survival tests were set for both the post-DGC, and the post-MACS sample; and motility was assessed at 0 and 24 hours. The DNA fragmentation was measured on the fresh, the post-DGC, and the post-MACS sample, by Halotech DNA SL.
**Results:** A significant difference was found in the DNA fragmentation percentage between the initial and the post-DGC samples (19.53±7.66 vs. 13.80±5.06; *P*-value=3,36x10-5), and between the post-DGC and the post-MACS samples (13.80±5.06 vs. 10.00±5.44; *P*-value=7.14x10-5). The reduction in the DNA fragmentation rate achieved between the initial and the final post-MACS sample was of almost 50%. No significant difference was found when the percentage of loss of motility between the 0 and the 24 hours was compared between the post-MACS sample (16.86±11.50) and the post-DGC control sample (23.77±13.92). The sperm motility index (SMI) for the sperm survival test was 0.88±0.17. There was no significant loss of sperm concentration after the elution through MACS columns.
**Conclusion:** The use of MACS does not have detrimental effects on the sperm survival rate or the quality of the semen sample, and they can be used to improve the outcome of assisted reproduction treatments as they are safe to use in the laboratory because of their good SMI. The combined use of DGC and MACS selects sperm with better DNA quality, than the use alone of DGC.

## P-15. Management of a simple blastocyst qualification system in embryo selection for transfer

**T. Puga-Torres[1], X. Blum-Rojas[1], M. Blum-Narváez[1]**

**Objective:** To corroborate the efficacy of a simple blastocyst classification system for embryo selection with good clinical results.
**Methods:** A retrospective evaluation of the quality of 122 blastocysts (day 5) obtained from ICSI procedures and transferred freshly on 66 patients was performed. The embryo score was performed by two embryologists, following the Sjoblom criteria (published by Richardson), through inter-observation of images of each embryo, without knowing the clinical result. The implantation rate was related to the classification system. The inter-operator results were evaluated by kappa statistical test.
**Results:** Sjoblom's blastocyst classification system simplifies their category alphabetically: A, B, C, Caviting and Compacting, with "A" being the best quality. Inter-operator evaluation showed a good level of agreement (0.62). The implantation rates obtained are described in Table 1. With these rates it was found that transfers with an A + 1 cavitating blastocyst would seem to have a better result; however, the number of transfers with these qualifications were too low to be considered.

| **Table 1:** Implatation rates obtained. | | | | | |
|---|---|---|---|---|---|
| Embryo Score | A+A | B+B | A+B | A+C | A+CAVITING |
| Implatation rate | 54.5 | 42.8 | 25 | 16.7 | 75.0 |

**Conclusion:** The Sjobom system for classification of blastocysts showed a good inter-observer correlation, allowing to simplify the score given to the embryos and good clinical results in the transfer of blastocysts with an A score.

## P-16. "Freeze-all" policy in poor diagnosis patients

**J. Cicare[1], P. Hovanyecz[1], P. Perfumo[1], L. Domenech[1], V. Ventura[1], M.V. Paz[1]**

**Objetive:** The "freeze-all" policy has emerged as an alternative to fresh embryo transfer to improve in vitro fertilization outcomes, since controlled ovarian stimulation may decrese endometrial receptivity. However, there is still controversy over which patients should be offered this strategy. The objective was to determine whether the frozen-thawed embryo transfer improves outcomes in patients with implantation failure and poor responders.
**Methods:** In this retrospective, observational study, we offered a frozen-thawed embryo transfer to 17 patients younger than 40 years-old who underwent ICSI procedures from June 2014 to December 2016. These patients had at least one previous fresh embryo transfer without having achieved an ongoing pregnancy. To poor responders, accumulation of at least two embryos on day 3 was offered before frozen-thawed embryo transfer. Pearson's chi-squared tests or Fisher's exact tests were used to compare clinical outcomes between fresh and frozen-thawed embryo transfers. Differences were considered significant if $P<0{,}05$.
**Results:** A total of 39 cycles were included: 22 in the fresh embryo transfer group and 17 in the freeze-all group. The results for fresh and freeze-all groups were: positive hCG levels 32% vs 65% ($P=0.085$); clinical pregnancy rate 9% vs 29% ($P=0.223$); ongoing pregnancy rate 0% vs 29% ($P=0.021$) and abortion rate 100% vs 0% ($P=0.095$), respectively.
**Conclusion:** Although no significant differences were found regarding positive hCG levels, clinical pregnancy or abortion, the ongoing pregnancy rate was significantly higher in the "freeze-all" group. This suggest that the "freeze-all" policy may improve the ongoing pregnancy rate in patients with implantation failure and poor responders, perhaps because it provides a more physiologic environment in which embryo transfer can occur. To extend these results it is necessary to increase the number of patients under study.

## P-17. Multinucleated embryo transfers and its impact on clinical outcomes

**P. Hovanyecz[1], J. Cicaré[1], L. Domenech[1], P. Perfumo[1], V. Ventura[1], M.V. Paz[1]**

**Objective:** To report clinical outcomes when multinucleated embryos are transferred only when there were no other suitable embryo after ICSI and to determine if the amount of nucleus in at least one blastomer of day-2 embryos (bi or multinucleated) could be associated with pregnancy rates.
**Methods:** A total of 26 cycles of patients younger than 40 years were included from January 2015 to December 2016. Multinucleation was evaluated on day-2 embryo after ICSI. All embryo transfers were performed on day 3 as the patients were all poor responders. β-human chorionic gonadotrophin subunit, clinical pregnancy, implantation and ongoing pregnancy rates were analized. Fisher's exact test was applied for statistical difference and the significance level was set at $P<0.05$.
**Results:** A total of 33 multinucleated embryos was transferred. 73% of embryo transfers (19/26) were single non-elective transfers. In double transfers, both embryos were multinucleated.The overall ongoing pregnancy rate was 19,23%. No significant differences were found in pregnancy rates between the transfer of bi or multinucleated embryos (Table 1).

**Table 1:** Clinical outcomes when bi and multinucleated embryos were transferred

| | Total | Binucle-ated | Multinu-cleated | *P*-value |
|---|---|---|---|---|
| Sub HCG+ | 30.7% (8/26) | 33.3% (5/15) | 27.3% (3/11) | 1.00 |
| Clinical pregnancy | 26.9% (7/26) | 26.7% (4/15) | 27.3% (3/11) | 1.00 |
| Ongoing pregnancy | 19.23% (5/26) | 20% (3/15) | 18.2% (2/11) | 1.00 |
| No of Newborn | 3 | 2 | 1 | |

**Conclusion:** In this study we report 3 healthy newborn when multinucleated embryos were transferred. Other two pregnancies are ongoing up to date. Aditionally, our results suggest that transferring a multinucleated embryo is not associated with lower pregnancy rates when it is compared with binucleated embryo transfers. Strong correlations have been demonstrated between multinucleation on early embryos and poor implantation/pregnancy rates. However, here we show that when not enough mononucleated embryos are available, it would be worth transferring embryos with more than one nuclei.

## P-18. Single versus group embryo cultures until day three of development

**C. Frautschi[1], A. D' Agostino[1], A. Dematteis[1], G. Estofan[1], D. Estofan[1], M. Hernández[1]**

**Objective:** To assess the effect of single versus group embryos culture until day three in the results obtained in ICSI cycles.
**Methods:** Design: prospective not randomized study. The study included patients up to 38 years old that performed cycles of ICSI between March and August 2015, having >5 mature oocytes injected with ejaculated spermatozoa. Patient allocation to Single Culture (n=58, 298 oocytes, 22

embryo transfers) or Group Culture (n=35, 473 oocytes, 37 embryo transfers) was performed after oocytes injection. Both treatments used 20 µL microdroplets under oil (Vitrolife), placing one vs three to four oocytes per droplet. Embryos were classified as good quality when showed expected number of symmetric blastomeres, <20% fragmentation and no multinucleation on day 2. One to two embryos were transferred on day 3. Mann-Whithney and Chi-square test were used to compare means and proportions. $P<0.05$ was considered significant.
**Results:** There were no statistically significant differences between Single and Group Culture groups in: number of oocytes injected (8.5±1.6 vs 8.2±1.9), normal fertilization rate (72% vs 73%), total embryos achieved and good quality embryos achieved per normal fertilized oocytes (67% vs 66%; and 43% vs 44%), number of embryos transferred (1.6±0.5 vs 1.8±0.4), biochemical and clinical pregnancy rate (45% vs 51%; and 41% vs 43%) and implantation rate (32% vs 31%).
**Conclusion:** Our findings show that until day three of development, group culture does not improve embryo quality or pregnancy rates compared with single culture. It remains to analyze both treatments in prolonged culture.

## P-19. Aneuploid screening results: first IVI Santiago report

**D. Machuca[1], E. Andrés[1], C. Troncoso[1], N. Al-Asmar[1], C. Rubio[1], M. Calonge[1]**

*[1]IVI Santiago, Chile.*

**Objective:** Perform a descriptive analysis of the results obtained after the implementation of the Preimplantation Genetic Diagnosis program at IVI Santiago.
**Methods:** Retrospective study. An exportation of the clinical data and a subsequent exploratory analysis was carried out. The study group consisted in all patients who underwent an ICSI with medical indication for preimplantation genetic screening (PGS) using the comparative genomic hybridization technique and next generation sequencing (NGS) between 2013 and 2016. A total of 420 cycles were analyzed in the study. All transfers were made in deferred cycles.
**Results:** Maternal age average was 41.3±3.3. A total of 295 PGS cycles were performed with 884 biopsied embryos; 40.78% were euploid and 59.22% were aneuploid. Chromosomes with a higher incidence of aneuploidies were: Chromosome 22 (11.77%), chromosome 16 (10.16%) and chromosome 21 (8.80%). The incidence of trisomy of chromosome 21 with absence of other aneuploidies was 1.83%. A total of 145 cycles of euploid embryo transfer were performed, with the following results: Clinical gestation rate (64.1%), implantation rate (56.4%) and clinical abortion rate (7.53%).
**Conclusion:** This report corresponds to the preliminary analysis of our data. The improvement observed in clinical results has contributed to the current trend of perform genetic diagnosis in patients with medical indication for PGS, being advanced maternal age the most frequent. Currently, in our center, screening is done by NGS. This has contributed to visibly increase the number of PGS cases given the lowest cost of the technique.

## P-20. Evaluation of seminal quality in patients with problems of fertility of the Human Reproduction Center of Lima (NACER)

**J.V. Diaz Pinillos[1], L. Burga Davila[1]**

*[1]Human Reproduction Center of Lima (NACER) - Peru.*

**Objective:** The aim of this study was evaluate the semen quality of patients with fertility problems who attended to NACER during the period May 2015 to May 2016.
**Methods:** The study population consisted of 150 individuals with aged between 27 and 70 years who underwent a seminal analysis, where seminal parameters was assessed such as volume, viscosity, pH, vitality, concentration and sperm morphology according to criteria of the World Health Organization (2010). Descriptive statistics, frequencies and media of the most frequent anomalies were calculated and compared with a normal condition in the most important seminal parameters in fertility. All analyzes were performed with a confidence level of 95% using SPSS version 23 for Windows 2010, also it was performed a frequency table that was used for get graphics in excel indicating the prevalence and incidence of alterations in semen analysis made.
**Results:** Of the total samples analyzed 50.6% showed at least one parameter altered, while 49.3% of cases fulfill the benchmarks set out in the 5th edition of the WHO manual (2010). The astenozoospermia was the anomaly most prevalent in the patients studied with a percentage of 28%, followed by oligozoospermia and hipospermia with a percentage of 19.3% each anomaly. In the macroscopic characteristics it found that 45 (30%) of cases evaluated presented abnormalities in consistency and 29% of the cases studied presented azoospermia.
**Conclusion:** The presence of alterations as astenozoospermia, oligozoospermia, hipospermia and Teratozoospermia reflects the decline in semen quality population that man has experienced in recent years.

## P-21. General public accessing ART: interlinking between public and private institutions

**J. Mas de Ayala[1], N. Barrera[1], J. Alciaturi[1], C. Bonelli[1], R. García[1], L. Cantú[1]**

**Objective:** In Uruguay, since 2015, infertility patients have the possibility to access public funding to cover their assisted reproductive technology (ART) procedures. The Ministry of Public Health has enabled several clinics to assess and medically treat these couples. The Centro de Esterilidad Montevideo is a private institution with more than 30 years of experience in this field, assisting an average of 350 couples per year before 2015. CEM was incorporated to this new public health program when it started two years ago, and has completed 70% of the cases that have been authorized. The objective was to evaluate how an increase in the number of cycles per year –more than doubling- affected the overall results in the in vitro fertilization laboratory.
**Methods:** Fresh cycles (own oocytes and egg-donation) completed between July 2015 and December 2016 were evaluated retrospectively.
**Results:** A total of 916 IVF/ICSI cycles were performed, 70% were totally or partially funded by public resources. The median age of patients was 38 years. The ongoing pregnancy rate was 30% in own-oocytes fresh cycles and 53.5% in egg-donation fresh cycles.
**Conclusion:** Patient´s average age did not differ from previous years. Despite there was not a significant increase in the number of technical staff personnel at the laboratory, pregnancy rates have not been altered and remain above average results in this region (Argentina-Paraguay-Uruguay). This efficient and competent coordination between private clinics and public agencies has positioned Uruguay as a pioneer country regarding public ART coverage.

## P-22. First reported of Double Ovarian Stimulation (DOS) in a crease cancer patient, an effective toll for fertility preservation

**D. Raso[1], B. Lotti[1], J. Barattini[1], G. Marconi[1], F. Neuspiller[1]**

[1]*IVI Buenos Aires - Argentina.*

**Introduction:** Life expectancy among cancer patients has increased dramatically due to the improvements in cancer treatments. The preservation of fertility becomes a common requirement for these patients prior to exposure to treatment´s gonadotoxic. Objective: To report the outcome of a Double Ovarian Stimulation (DOS) during the folicular and luteal phases in the same menstrual cycle of a breast cancer patient.
**Case report:** Patient: A 41-year-old woman with breast cancer performing DOS in order to preserve fertility prior to chemotherapy. Intervention: Double Ovarian Stimulation (DOS). Main Outcome Measure: number of oocytes. Results: Following DOS a total of 12 MII oocytes were obtained and vitrified.
**Comments:** DOS during a cycle is a safe and feasible technique for attempting to preserve female fertility in emergency situations like cancer patients, before gonadotoxic chemoterapic treatment takes place.

## P-23. Endometrial Receptivity Analysis: a useful tool for personalized embryo transfer in patients with related implantation failure

**B. Lotti[1], D. Raso[1], F. D'Atri[1], N. Fernández Peri[1], F. Neuspiller[1]**

[1]*IVI Buenos Aires - Argentina.*

**Objective:** To assess the presence of an alteration in the window of implantation in patients with repeated implantation failure by endometrial receptivity analysis (ERA). To asses pregnancy rate and implantation rate in patients with repeated implantation failure and nonreceptive endometrium by Endometrial Receptivity Analysis who have performed a personalized embryo transfer.
**Methods:** Retrospective, analytic, observational and transversal study that included patients who performed Endometrial Receptivity Analysis in IVI Buenos Aires between December 2013 and February 2017.
**Results:** Fifty-four patients with repeated implantation failure who performed Endometrial Receptivity Analysis were included. Thirty-nine patients had a receptive result (72.22%) and fifteen patients had a nonreceptive result (27.78%). The last one were divided into pre-receptive (86.67%) and post-receptive (13.33%). Seven patients with a nonoreceptive status have had personalized embryo transfer, with a pregnancy rate of 71.43% and an implantation rate of 55.56%
**Conclusion:** Endometrial Receptivity Analysis is a tool which allows to perform a personalized embryo transfer taking into account the endometrial status. It becomes an objective test which is able to identify displacements in the window of implantation in patients with repeated implantation failure, increasing pregnancy and implantation rates.

## P-24. Non-treated male accessory gland in section syndrome (MAGI-S) may impair semen quality

**C. Avendaño[1,2,3,4], D. Ferrer[1,2], J. Ojeda[5], G. Gallardo[1,2], A. Juárez Villanueva[1,2]**

**Objective:** There are some controversies about the negative effect of male genitourinary tract infections on fertility. MAGI-S is a combination of at least two or more of the following seminal alterations: viscosity>2cm, pH>8.5, round cells>5x106/mL, leucocyte>2x105/mL, sperm antibodies>10% or abnormal seminal biochemistry. Generally, the usefulness of these parameters is underestimated in the routine fertility potential evaluation. The aim of this study was to evaluate the impact of MAGI-S on male fertility potential/semen quality in men with or without treatment of the infection.
**Methods:** Semen was collected after 2-4 days of sexual abstinence. Volume, pH, viscosity, concentration, motility, morphology, vitality, sperm antibodies and seminal biochemistry were analyzed. Semen samples from each patient were analyzed at two time points: t0, before treatment; t1: at least 6 months after t0. Three groups were established according to the lab diagnosis and treatment; G1: MAGI without treatment; G2: MAGI with treatment; G3: non-MAGI. Intra-patient variability was compared by Mann-Witney U test.
**Results:** The mean period between t0 and t1 was comparable for all groups (1.45 years +/- 0.5; P>0.05). By comparing t1 vs t0, a significant decrease of sperm quality was found in G1, whereas G2 showed a significant increase. No significant difference in G3 was found. The results are resumed in the table below.

| Parameter | Group | Media ± Standard Error $T_0$ | Media ± Standard Error $T_1$ | *P* value |
|---|---|---|---|---|
| Concentration (x10[6]/mL) | 1 (n:37) | 86.9±12.4 | 80.7±10.4 | >0.05 |
| | 2 (n:24) | 100.7±18.4 | 120.6±16.3 | >0.05 |
| | 3 (n:15) | 82.4±13.4 | 92.3±13.4 | >0.05 |
| Total motility (%) | 1 (n:37) | 62.9±2.9 | 54.6±2.9 | <0.05 |
| | 2 (n:24) | 56.0±3.3 | 74.0±2.1 | <0.001 |
| | 3 (n:15) | 69.1±3.9 | 71.3±3.7 | >0.05 |
| Morphology (%) | 1 (n:37) | 4.4±0.5 | 2,1±0.3 | <0.001 |
| | 2 (n:24) | 2.3±0.4 | 4.9±0.5 | <0.001 |
| | 3 (n:15) | 4.8±0.8 | 4.5±0.5 | >0.05 |
| Vitality (%) | 1 (n:37) | 80.7±2.1 | 72.3±2.4 | <0.001 |
| | 2 (n:24) | 82.4±1.4 | 85.3±1.0 | <0.05 |
| | 3 (n:15) | 84.1±1.8 | 83.4±1.6 | >0.05 |

**Conclusion:** Our results show that MAGI-S may impair sperm function and decrease male fertility potential. We postulate that the complete semen analysis is a valuable tool to prevent deleterious effect of accessory gland infections on seminal quality.

## P-25. Routinely freezing al blastocyst and transfer in a non-gonadotrophin stimulated cycle as a strategy to increase pregnancy outcomes in IVF

**A. Abdala[1], M. Nuñez[2], E. Gómez Passanante[1], F. Raffo[1], S. Provenzano[1], J. Singla[1]**

**Objective:** To evaluate whether cryopreservation of all embryos reaching the blastocyst stage and transfer in a non-gonadotrophin stimulated cycle increase clinical pregnancy rates per embryo transfer cycle.
**Method:** A retrospective cohort observational study was carried out between 2014 and 2016. All women between 27 and 41 years-old with own fresh oocytes inseminated by ICSI or conventional IVF that had realized at least one fresh blastocysts transfer or had cryopreserved all blastocysts for medical or personal reasons and had realized a subsequent frozen-thawed embryo transfer in a non-gonadotrophin stimulated cycle were analyzed. Patients with severe male factor or cryopreserved semen were excluded
**Results:** 85 women with a mean age of 35, 29±3, 75 were included. Patients were divided in 2 groups: 54 fresh blastocyst transfers and 31 frozen-thawed blastocyst transfers. There were no statistically significant difference in mean age, global fertilization rates, mature oocytes rates and number of embryos transferred per patient (35.52±3.71 and 34.81±3.85, *P*=0.4234; 79% and 83.8%, *P*=0.802; 78.5% and 81.8%, *P*=0.802; 2.03±0.26 and 1.89±0.42, *P*=0.550, for fresh and frozen blastocysts). Clinical pregnancy rate for frozen-thawed blastocyst transferred was significant higher compared with fresh blastocysts transferred (59.3% vs 25.9%, *P*=0.0031; Odds Ratio: 4.17; 95%, CI: 1.61-10.78, *P*= 0.0031).
**Conclusion:** Clinical pregnancy rates increase with frozen-thawed blastocysts transfers demonstrating that total blastocysts cryopreservation could be a routinely strategy for increasing IVF outcomes.

## P-26. The endometrial from women with recurrent implantation failure exhibit abnormal expression of complement regulatory proteins. A promising immunologic marker of endometrial receptivity

**A. Palomino[1], C. Aguirre[1], F. Argandoña[1], K. Sequeira[1], L. Devoto[1]**

**Objective:** The control of maternal immunologic response is critical to prevent embryo rejection. The complement activation must be restrained within embryo-maternal environment to ensure embryo survival and successful pregnancy development. Complement system proteins are expressed in endometrium during embryo receptivity phase. The mechanism involved in the recurrent implantation failure (RIF) in assisted reproduction remains unknown. Objective: To assess the expression of complement regulatory proteins: decay accelerating factor (DAF), CD46 and CD59 in the endometria of women with recurrent implantation failure (RIF).
**Methods:** RIF was defined as the failure of embryo implantation after three in vitro fertilization (IVF) cycles in which one or two morphological high-grade embryos were transferred. Endometrial biopsies from 35 patients with RIF were obtained five or six days after progesterone administration during mock hormonal endometrial preparation cycle for embryo transfer. Endometrial samples from 24 fertile women obtained during mid-secretory phase were included as controls. Proteins were localized by immunohistochemistry and their relative expression determined using histological score (Hscore). The mRNA expression was determined by qRT-PCR. To compare the findings non-parametric Mann-Whitney statistical test was applied.
**Results:** According to Hscore and mRNA levels, DAF expression was significantly low in samples from RIF's patients (*P*<0.05).
**Conclusion:** Reduced DAF expression may lead to uncontrolled complement activation within embryo-endometrial environment risking embryo survival that potentially explains RIF in assisted reproduction cycles. DAF expression emerges a hopeful marker of endometrial receptivity to guide embryo transfer in assisted reproductive cycles
**Support by:** Fondecyt 1140688

## P-27. A preliminary retrospective study on sperm banking. The Uruguayan experience

**R. Ordoqui[1], N. Barrera[1], J.M. Montes[1], M. Canepa[1], C. Bonelli[1], C. Surka[1], A. Torrens[1], L. Cantú[1], S.S. Du Plessis[2]**

**Objective:** Sperm cryopreservation is a biotechnology widely used in andrology laboratories. Fertilab, the only sperm bank in Uruguay, has already more than 29 years of experience in employing this technology. Objective: To illustrate the status of homologous sperm cryopreservation in Uruguay.
**Methods:** 324 semen samples cryobanked between 2013 and 2015 were analyzed. Reasons for sperm banking, patient age, pre-freeze and post-thaw semen parameters and recovery rate was reviewed.
**Results:** In most cases samples were stored due to patients undergoing either assisted reproductive technology (ART) treatment (n=190 or 58,64%) or because of oncological causes (n=113 or 34,88%). The median age of bankers were 34 years. Among the Oncological group 61.95% (n=70) of cases were diagnosed with testicular cancer. Medians of semen parameters for both groups were above the lower reference limits according to 2010 WHO guidelines. In fresh samples a significant difference were observed in progressive motility (ART=47% vs. Oncological=56%; $P<0.05$). After thawing, total motility (27% vs. 32%, $P<0.05$), progressive motility (19% vs. 22%; $P<0.05$) and vitality (48 vs. 56%; $P<0.05$) differed significantly between the ART and Oncological bankers.
**Conclusion:** The primary reason for sperm cryopreservation is fertility treatment. Sperm banking for this reason is usually short term and help to reduce men´s anxiety and ensure a backup sperm sample on the day of ART. Surprisingly, the oncological group showed semen parameters considerably close to normal. It is evident that semen banking can be performed successfully in Uruguay and outcomes are on par with international standards.

## P-28. Maternal age and aneuploidies by Next Generation Sequencing (NGS)

**P. Villanueva[1,2], L. Noriega-Hoces[1,2], F. Vizcarra[1], J. Portella[1,2], R. López[1,3], A. Villalobos[2], L. Guzmán[1,2,3]**

**Objective:** In vitro fertilization (IVF) has become a common practice nowadays. In order to increase pregnancy rates; several techniques to identify the higher likelihood embryo implantation have become a routine. Some of this advance methods are genetic studies, like NGS, allows us to detect abnormalities with better sensibility and specificity. Objective: To determine the rate of euploidy, aneuploidy and mosaicism in embryos according to the maternal age.
**Methods:** From January 2016 to December 2016; three hundred and seven IVF cycles generated nine hundred thirty three blastocyst. All blastocyst were biopsied and referred to a reference center (Reprogenetics Latinoamérica) for Preimplantation genetic screening (PGS). DNA from trophoectoderm biopsy was amplified with Sureplex; hr-NGS sequencing was performed in a MiSeq Illumina (VeriSeq PGS assay). NGS data was analyzed by Bluefuse Multi Analysis (Ilumina). Embryos with one or more chromosomic aneuploidies were classified as aneuploids; embryos with one mosaicism were classified as mosaics. Descriptive statistics, parametric and non-parametric bivariate analyses (ANOVA and Kruskal Wallis), and, generalized linear models (GLM) were evaluated, considering $P\leq0.05$ as significant. All of these analyses were performed in Stata v.14. Embryo with a single or multiple aneuploidy with/without mosaicism was classified as aneuploid. Embryos with only mosaicism were classified as mosaic. Study population was classified according to the origen of the oocytes: egg donor (N=55) and IVF patients (N=252). IVF patients were sub-classified in five age categories according to SART criteria (<35; 35-37y; 38-40y; 41-42y; <42y).
**Results:** Our results indicated that aneuploidy increased with maternal age. In the egg donor group aneuploidy rate was 35.1±26.1 and the frequency in the IVF patient group was in concordance with SART criteria, aneuploidy rate per group were as follow: 40.4%±28; 39.9%±34.9; 62.7%±37.4; 71.4%±31.6 and 70.2%±35.9 respectively ($P<0.001$). On the contrary, mosaicism in egg donor group was 15.5%±20 and remains constant among the IVF patient groups 13.2%±22.5; 10.4%±23.5; 5.9%±13.5; 7.3%±16.4 and 10.4%±21.6 respectively ($P=0.442$). GLM between age and co-variates, resulted in strong significant association for euploidy and aneuploidy. Scatterplot for euploidy showed a negative tendency between IVF women; the more age the patients have, the less proportion of normal embryos.
**Conclusion:** NGS results confirmed the association between maternal age and aneuploidies. However is necessary evaluating other risk factors in prospective studies to determine their influence.

## P-29. Oocyte vitrification in a donation program

**A. Mata[1], M. Pérez[1], C. Maino[1], J. Heredia[1], J. Pérez Alzaa[1]**

[1]*Fundación Fecundart, Córdoba, Argentina.*

**Objective:** To show the results of a three-year experience of an egg donation program using cryopreserved oocytes.
**Methods:** The data of 89 egg donation cycles using vitrified oocytes (Group A=79 recipients) were analyzed retrospectively and compared with 22 fresh donation cycles (Group B=22 recipients). Three hundred and twenty four oocytes from 54 donors, vitrified using the Cryotop method were thawed between January 2014 and December 2016. After warming, surviving oocytes were inseminated by ICSI and cultured until transfer (days 3 or 5-6).
**Results:** In the vitrified oocyte group (A) the survival rate after warming was 84.8%.
No statistical differences were found when comparing groups A and B with respect to mean number of metaphase II oocytes (MII) allocated to each recipient (3.5 vs. 3.6); mean age (25.9 vs. 27.4 years) and BMI (24.9 vs. 23.3) of donors, or number of oocytes obtained after follicle aspiration (11.7 vs. 10.3). Fertilization rates (79.6% vs. 81.3%) and mean number of transfered embryos (1.8 vs. 1.7) were also similar.
Pregnancy (44.6% vs. 40.0%), implantation (27.1% vs. 26.5%) and miscarriage rates (16.7% vs. 11.1%) were resembling in both groups (A vs. B).
**Conclusion:** Even though oocyte vitrification has been successfully used world- wide for the past years, most of egg donation cycles in Latin America use fresh oocytes. The acceptable clinical results together with the advantages over fresh oocyte donation (no need of donor-recipient synchronization plus a more efficient distribution of the available oocytes) make oocyte banking a valuable approach in egg donation programs.

## P-30. Mosaic embryo transfer after oocyte in vitro maturation in combination with non-invasive prenatal test (NIPT) - First report of a live birth

**N. Inoue[1], R. Lopez[2], A. Delgado[3], D. Nuñez[1], J. Portella[1], L. Noriega-Hoces[1,3], L. Guzmán[1,2]**

**Introduction:** Oocyte in vitro maturation (IVM) is not considered as a well-established technique in IVF Laboratories. This is due to a lower pregnancy rates published. However in the last few years, reports have shown similar pregnancy rates compared to conventional IVF techniques. The current report describes the first life birth after an IVM treatment in combination with PGS.
**Case Report:** Embryos were cultured until blastocyst stage, resulting in a euploid embryo after aCGH diagnostic. However, the NGS re-analysis showed that embryo was a mosaic for chromosome 13 and 21. Nevertheless, pregnancy ultrasound scans and non-invasive prenatal test (NIPT – Verifi - Illumina) indicated a normal fetus development. Finally, a healthy baby was born after 38 weeks, its weight was 4480gr, head circumference 36 cm and total length of 51 cm. To confirm that the baby was chromosomically normal a NGS test was performed in buccal cells, a normal profile was obtained.
**Comments:** Our finding confirmed that mosaic embryo transfer would bring a healthy offspring.

## P-31. Sequential aneuploidies in human blastocyst and their relation with maternal age

**R.A. Sánchez Usabiaga[1], C. Regeyra Edelman[1], C. Duran Montaño[1], E.G. Ramirez Rivera[1], E. Gonzalez Becerra[1]**

[1]*Médica Fértil - Mexico.*

**Objective:** Determine the incidence of blastocyst with segmental aneuploidies (deletion and duplication) and their relationship with maternal age.
**Methods:** We included 615 biopsies of trophectoderm from blastocysts (day 5 and day 6) of development, which were submitted to a reference genetic laboratory (Natera; San carlos,CA) for single nucleotide polymorphism array of the 24 chromosomes. The results were analyzed under the algorithm termed "Parental Support", the key insight of this algorithm is that copy number determination in embryonic DNA is enhanced if supplemented with parental genetic data. This method allows determine blastocyst chromosomal copy numbers and segmental aneuploidies.
**Results:** They were divided into four maternal age ranges: "<35" (n=238), "35 to 37" (n=143), "38 to 40" (n=163) and "≥41" (n=71). The 50.3% of the total blastocysts were aneuploidy, of which 12.3% presented segmental aneuploidies (maximum value of 20% in <35 and a minimum of 2% in ≥41), and 5.03% segmental aneuploidies combined with other types of aneuploidies (Maximum value of 9% in <35 and minimum of 0% in ≥41), in both cases the rate of segmental aneuploidies decreased with increasing maternal age, presenting a linear relation of R2> 0.97.
**Conclusion:** Segmental aneuploidies affect a large number of blastocysts, being inversely proportional to maternal age. Segmental aneuploidies represent an area of research that will help us to know its clinical relevance in human reproduction and can be considered within the genetic counseling in preimplantation genetic screening.
Key words: Segmental aneuploidies, In vitro fertilization, Blastocyst, preimplantation genetic screening.

## P-32. Possible influence of the different shipment types on the viability of vitrified oocytes

**B. López[1,2], E. Criado[1,2]**

[1]*FIV Marbella – Ovobank, Spain.*
[2]*Ovobank, Spain.*

**Objective:** To analyse the possible influence of different shipment types on the viability of vitrified oocytes
**Methods:** Oocytes at methaphase II from donors aged 18-31 were used. They were collected via follicular puncture 36 hours after trigger, then vitrified following Kitazato protocol (Cryotop) and stored in tanks (liquid nitrogen) afterwards at Ovobank laboratory (FIV Marbella, Spain). Vitrified oocytes were shipped into Dry Shipper Dewars containers during the research, from November 2014 until September 2016, in two types of shipments: Standard air delivery by plane: Conventional Cargo Company and Lab to Lab: An ovobank staff member is personally in charge of the door-to-door delivery (Air or land). The survival data from every centre have been analysed and they have been classified by their shipment type in an Excel table for subsequent analysis.
**Results:** 5336 shipped oocytes, 5262 thawed have been reported: 2278 thawed oocytes were sent via standard air delivery, and 2984 thawed oocytes were sent via Lab to Lab. The survival rate is analysed according to the type of shipment and it is significantly higher in Lab to Lab deliveries than in standard delivery (74,9 vs. 70,5%).
**Conclusion:** Data shows that oocytes may be damaged if they are shipped via standard carrier. This is probably due to the container handling, the use of commercial airlines, etc. Due to the constant flux of oocytes shipments by Ovobank, we are constantly receiving results, so more studies are needed for researching, analysing and going in depth on the influence of the different shipment types on the viability of vitrified oocytes.

## P-33. Delayed blastocyst formation and its influence on frozen-trowed transfer. Day 5 vs Day 6

M.V. Paz[1], J. Cicaré[1], P. Hovanyecz[1], L. Domenech[1], P. Perfumo[1], V. Ventura[1]

**Objective:** To compare the pregnancy rates between good quality blastocysts vitrified on day 6 versus blastocysts vitrified on day 5 after fertilization.
**Methods:** In this retrospective study 134 freeze-thaw cycles of blastocysts were vitrified on either day 5 or day 6 and transferred between January 2015 and December 2016. Only patients younger than 40 years old, with at least one good quality blastocyst (≥3BB) for transfer were included. All the embryos were thawed and transferred on day 5 of progesterone treatment in hormonally prepared cycles. Clinical outcomes were compared between both groups. Student's t test and chi-squared test were used for comparison of variables.
**Results:** 97 cycles included blastocysts vitrified on day 5 and 37 cycles on day 6. The results for day-5 and day-6 freeze-thaw cycles groups are shown on Table 1. No significant differences were found in clinical outcomes between both groups.

**Table 1:** Clinical outcomes of frozen cycles between blastocysts vitrified on day 5 and on day 6.

| | Blastocysts vitrified on day 5 | Blastocysts vitrified on day 6 | *P* |
|---|---|---|---|
| Cycles (n) | 97 | 37 | |
| Nº embryo transferred (mean) | 1.76±0.42 | 1.76±0.43 | >0,99 |
| HCG positive | 59.80% (58/97) | 54.10% (20/37) | 0.85 |
| Clinical pregnancy rate | 46.40% (45/97) | 40.50% (15/37) | 0.68 |
| Implantation rate | 39.53% (68/172) | 26.15% (17/65) | 0.07 |
| Ongoing pregnancy rate at 20 week of gestation | 40.20% (39/97) | 35.10% (13/37) | 0.73 |

**Conclusions:** Day 5 and day 6 blastocysts, having the same morphological quality at the time of freezing, showed no difference regarding clinical pregnancy and ongoing pregnancy rates. It is noteworthy that day-6 blastocysts transfers were performed on day 5 endometrial preparation. It could be speculated that endometrial-embryonic synchronization could be more relevant than the rate of blastocyst development in treatment outcomes.

## P-34. Gender differences in dealing with assisted reproduction treatment: psychological and social reaction in men and women

H. P. Lopes[1], C. Avelar[2]

**Objective:** Increasing numbers of children are conceived through assisted reproduction techniques (ART). Using a comprehensive approach, the present research seeks to contribute to the understanding of gender differences in coping with infertility and in dealing with ART treatment.
**Methods:** Our sample consisted of 40 couples aged to 24 to 43 and the data were collected through semi-structured interviews.
**Results:** In the studied sample, gender differences existed not only in experiences of infertility but also in dealing with ART treatment. Responses to stress caused by infertility were stronger in women than men. For women the central aspect of infertility was the desire for a child while for men the central aspect was the social obligation imposed to fulfill the male role. To understand the complexity of infertility, the psychological and social dimension in how men and women cope with the treatment was the central aim of the present study. The women expressed more strongly emotion reaction to their infertility than the men. They received more social support than their partners, who experienced the fulfilment of the male role as well as the social role to become a parent.
**Conclusion:** These findings emphasize the importance of psychological support during ART treatment oriented to the gender difference, recognizing, naming and intervening to work on these differences.

## P-35. Results of intrauterine insemination versus intracytoplasmic sperm injection in patients between 40 and 43 years

C. Lucini[1], A. Pasqualini[1], Y. Aza[1], C. Piscicelli[1]

**Objective:** To compare pregnancy rates between Intrauterine Insemination (IUI) and Intracytoplasmic Sperm Injection (ICSI) in patients ≥ 40 and ≤43 years.
**Methods:** Retrospective and observational study. Data was obtained from medical histories of patients ≥ 40 and ≤43 years, who underwent intrauterine insemination (up to 2 prior) or first intracytoplasmic injection, between 2010 and 2017 (January).
**Results:**

Patients

| CHARACTERISTICS | IUI | ICSI | *P* |
|---|---|---|---|
| n | 508 | 562 | |
| Mean age (years) | 41.25±1.08 | 41.13±1.02 | 0.062 |
| Primary Infertility | 61.22% | 55.5% | 0.059 |
| Secondary Infertility | 38.73% | 44.5% | 0.059 |
| Infertility duration | 2.19 years ±5.17 | 3.57 years ±8.04 | 0.008 |
| Cervical Factor | 2.16% | - | 0.01 |
| Unexplained infertility | 8.85% | 3.9% | 0.01 |
| Diminished ovarian reserve | 31.69% | 33% | 0.669 |
| Male infertility | 5.31% | 10.6% | 0.01 |
| Endometriosis | 1.18% | 0.5% | 0.247 |
| Multiple causes | 30.11% | 35.4% | 0.066 |

Pregnancy rates per cycle

| | IUI (n =508) | ICSI (n=562) | *P* |
|---|---|---|---|
| All patients (40-43) | 6.29% (32/508) | 16.9% (95/562) | 0.01 |
| 40 years | 9.09% (12/132) | 25.3% (48/189) | 0.01 |
| 41 years | 4.10% (6/146) | 15.9% (29/182) | 0.01 |
| 42 years | 8.10% (9/111) | 10.16% (12/118) | 0.589 |
| 43 years | 3.37% (3/89) | 8.2% (6/73) | 0.180 |

Pregnancy Outcomes

| | IIU( n=32) | ICSI(n=95) | *P* |
|---|---|---|---|
| Clinical pregnancy | 31.25% (10/32) | 43.15% (41/95) | 0.3271 |
| Miscarriage | 12.5% (4/32) | 18.9% (18/95) | 0.5731 |
| Chemical Miscarriage | 15.6% (5/32) | 29.47% (28/95) | 0.189 |
| Ectopic pregnancy | 3.12% (1/32) | 2.10% (2/95) | 1 |
| Unknown | 37.7% (12/32) | 5.2% (5/95) | |
| Twins | 0 | 1.05% (1/95) | 1 |

**Conclusion:** The pregnancy rates between intracytoplasmic injection and intrauterine insemination are not statistically significant in women aged 42 and older. Prospective and randomized studies are needed to arrive at better conclusions.

## P-36. Psychopathological status, previous infertility diagnosis and failed fertility treatments in members of infertile couples

**L. Jurkowski[1], P. Balbi[1], N. Bossi[1], C. Granados[1], E. Polak de Fried[1]**

**Objective:** To describe and compare the psychopathological status of members of infertile couples with previous infertility diagnosis and previous failed fertility treatments at their first appointment at a clinic.
**Methods:** Observational and descriptive study. Sample: women (n=50) and men (n=48) (Age: x̄ 38.59 +/-6.57 years) completed the Symptom Checklist-90-R a test that assesses psychopathological status (Derogatis, 1994). The psychopathological status was analyzed by the following variables: A=Sample was compared by gender; B=Sample was compared by previous infertility diagnosis and C=Sample was compared by previous failed fertility treatments. Statistical analysis was made with Mann-Whitney U test, with the SPSS20. Statistical significance = *P*< 0.05.
**Results:** A=Men and women obtained higher scores in the following SCL-90-R dimensions: Somatization, Depression and Obsessive-compulsive. Somatization was higher in men (X47.06+/-9.61) and depression was higher in women (X46.9+/-12.07). B=Participants with previous infertility diagnosis presented higher scores of Phobic Anxiety than those without one (X42.07+/-11.23 versus X34.09+/-9.08 respectively, *P*=0.009). C=Subjects with previous failed fertility treatments obtained higher scores of Depression than those for whom it was the first time (X47.59 +/- 9.48 versus X43.03 +/-11.82 respectively, *P*=0.002).
**Conclusion:** Both male and female had the same psychopathological dimensions affected. Although Somatization was higher in men and Depression in women, this difference was not statistically significant. Previous infertility diagnosis relates to Fobic Anxiety and failed fertility treatments to depression symptomatology in members of infertile couples. This could be a first approach to develop personalized treatments with specific interventions to increase quality of life and fertility treatment adherence.

## P-37. Embryo transfer quality evaluation performed by clinicians and embryologists. Pregnancy rate impact

**J. Notrica[1], C. Granados[1], N. Bossi[1], E. Polak de Fried[1]**

**Objective:** To evaluate the coincidence or not of the clinical and laboratory appreciation of embryo transfers and its correspondence with pregnancy rate (PR).
**Methods:** In the same period of time, 151 embryo transfers were performed and evaluated by three physicians (G) and one embryologist (L). A comparison of these evaluations was made. All embryo transfers were performed with ultrasound guidance. Based on the presence or absence of mucus, blood, time of duration, type of catheter and use of shirt, the embryologist classified in L1, L2 and L3.
L1: absence of mucus or blood, use of soft catheter, without shirt, normal duration.
L2: presence of any of these parameters.
L3: combination of two or more parameters.
Medical embryo transfer quality classification was based on the degree of difficulty or not to cross cervical channel with the elected catheter loaded: G1, G2 and G3.
G1: no difficulty
G2: moderate difficulty (use of shirt)
G3: severe difficulty (change of catheter, retained embryo, presence of blood, longer than normal)
**Results:**
- 131/151 (86.7%) G1 while 79/151 L1 (52.3%).
- 45/131 G1 (34.3%) while 26/79 L1 (32.9%) got pregnant.
- 21/151 G2 (13.9%) and 41/151 L2 (27.15%).
- 8/21 (38%) G2 and 16/41 (39 %) L2 got pregnant.
- There was no G3 while 31/151(20,5%) were L3.
- 11/31 (35%) L3 got pregnant.

**Conclusions:** There were differences between the evaluations performed by the clinicians and the laboratory. According to the findings of this presentation we can not suggest that embryo transfer quality, as a unique factor, has an adverse impact on pregnancy rate.

## P-38. Sperm DNA fragmentation (Tunel) in the evaluation of male infertility a new cut-off value

**C. Alvarez Sedó[1], M. Bilinski[1], D. Lorenzi[1], M. Miguens[1], C. Borghi[1], S. Papier[1]**

**Objective:** The aim of this study was to establish the cutoff value, sensitivity and specificity of the TUNEL assay (sperm DNA fragmentation), and studying the distribution of the DNA damage in a fertile and infertile men population evaluated in an andrology laboratory.
**Methods:** For the present study, 48 infertile men (2014-2016) were enrolled. All infertile patients for male factor infertility were studied. A higher population of infertile men was initially enrolled, but they were excluded because a female factor was found (>37 y/o, poor ovarian response, tuboperitoneal factor, polycystic ovarian syndrome, altered karyotype). Thirty-two healthy male volunteers (controls) with proven fertility (<12 months) were selected. Samples were collected by masturbation. Semen analysis and motile sperm were selected using swim-up according to the procedures established by the WHO (2010). Sperm DNA fragmentation was assessed over motile sperm by TUNEL assay. T-test and ROC curve analysis were used for statistical analysis.
**Results:** Sperm motility, strict morphology and DNA fragmentation were significantly higher in patients compared to controls ($P<0.05$). The ROC curve analysis reported that the cutoff value was 15% (AUC: 0.78), with 53% of sensitivity and 100% of specificity. Detailed results are shown in the following table.

| | FERTILE | INFERTILE | *P* |
|---|---|---|---|
| Age | 33.2±4.7 | 36.5±5.2 | 0.99 |
| Sperm Concentration (mill/mL) | 58.1±20.4 | 45.5±40.6 | 0.22 |
| Progressive motility (%) | 59.2±16.1 | 47.8±19.8 | 0.01 |
| Strict morphology (%) | 13.1±2.5 | 6.5±3.8 | 0.01 |
| PMN (mill/mL) | 0.6±0.01 | 1.8±0.2 | 0.99 |
| TUNEL | 7.4±2.8 | 17.8±10.8 | 0.01 |

**Conclusion:** We have reported the values and reference ranges for the TUNEL assay (sperm DNA fragmentation) in semen samples of fertile and infertile men. A cutoff value of 15% with 100% specificity can differentiate infertile men with DNA damage of fertile men. TUNEL test for the evaluation of sperm DNA fragmentation is a useful test in the evaluation of male factor.

## P-39. Blastocyst mitochondrial DNA quantification and its association with female age, aneuploidy and implantation

**C. Alvarez Sedó[1], M. Bilinski[1], D. Lorenzi[1], M. Fabbro[1], F. Nodar[1], S. Papier[1]**

**Objective:** The aim of this study was to examine the relationship between human blastocyst mtDNA content, female patient age, embryo chromosome status, viability and implantation potential.
**Methods:** For the present study, 190 blastocysts (TE) (2016-2017) were studied. Blastocysts were produced by 64 couples of an average female age of 35 years (21–43 y/o). TE samples underwent NGS (illumina) analysis in order for chromosome constitution assessment and mtDNA quantification. Reads were aligned to the human genome hg19 for MiSeq sequencing runs. After alignment, unmapped reads, duplicate reads, reads with low mapping scores were removed. After alignment, genome Coverage Bed files were generated using BEDTools and the fraction of total sequenced bases that aligned to the mitochondrial genome relative to the nuclear genome was calculated. mtDNA was analyzed regarding the embryo aneuploidy, maternal age and implantation. T-test analysis was used for statistical analysis.
**Results:** Seventy-two embryos were considered as euploid (41%) and 92 embryos were aneuploid (59%). The amount of mtDNA was significantly higher in aneuploid embryos (0.065 vs. 0.040) ($P<0.05$). However, the amount of mtDNA were not different between embryos from young and older women (≤35 y/o vs >35 y/o) (0.051 vs. 0.054). When euploid embryos were transferred (27 cycles), a significant difference of mtDNA were found in embryos that implant regarding to those than not (0.030 vs. 0.048), 0.046 was the maximum amount of mtDNA found in embryos with ongoing pregnancy.
**Conclusion:** The relationship between mtDNA quantity and the potential of an embryo to successfully become a pregnancy was found. This support the proposal of mtDNA assessment for embryo selection in order to have the highest ability to lead to healthy pregnancies.

## P-40. Fetal fraction in maternal plasma could be affected by fetal aneuploidy and maternal weight experience in 1600 NIPT cases

**C. Alvarez Sedó[1], P. Nicotra[1], G. Fiszbajn[1], S. Kopelman[1], S. Papier[1], J. Hamer[1]**

**Objective:** The objective of this study was to analyze the different variables (maternal or fetal) that may be affecting the FF levels in the NIPT studies.
**Methods:** For the present study 1683 NIPT test were considered (2013-2016). For the FF analysis, patients were group accordingly to: gestational age, maternal height, weight, BMI, and fetal chromosome aneuploidies. Pearson and Jonckheere-Terpstra test were performed for statistical analysis.
**Results:** FF values had a normal distribution. The main value of FF was: 8.9±3.8. The 34% of the patients had 10-10.6 weeks of gestation, followed by 9-9.6 weeks (24%). However, there were no differences between FF values and gestational age. FF was inversely correlated with maternal weight (-0.44), height (-0.30) and BMI (-0.46) ($P$<0.001). In patients with a failed result in the first extraction (3.6%), the FF was 2.6%. Nonetheless, 57% of them had a result in the second extraction with a FF of 6.8%. The final percentage of NIPT failed results in our population was 1.6%. When FF was <2.6%, 100% of the patients had a redraw, however, when FF>2.6% only 2% needed a redraw. The average fetal fraction of samples with euploid result (1513) and trisomy 21 were 9.2% and 9.6%, respectively. In contrast, the median fetal fraction values for samples with trisomy's 18 and 13 were 6.8% and 4.8%, respectively.
**Conclusion:** The probability of having a NIPT failed result is low. The lack of results is strongly influenced when the FF <2.6%, also, is negatively correlated with weight, height and BMI. The fetal fraction of samples with trisomy 21 NIPT result is comparable to that samples with euploid result, but T18 and 13 had a lower value.

## P-41. Genetic carrier screening in sperm and oocyte donors

**C. Alvarez Sedó[1], A. Quinteiro Retamar[1], C. Borghi[1], P. De Carvalho[1], F. Nodar[1], S. Papier[1]**

**Objective:** The aim of this study was to establish the prevalence of genetic carriers (250 diseases) in our sperm and oocyte donor's population.
**Methods:** This a retrospective prevalence study. Materials and Methods: Twenty-nine (male) (21-39 y/o) and 460 (female) (21-33 y/o) donors were included (2013-2016). All donor signed an informed consent to participate in this study. All patients were evaluated by a clinical geneticist, in order to detect relevant family history (before and after the study). A blood sample was taken, and DNA was extracted. Dried DNA was sent to Recombine Laboratory (NJ, USA). A genomic platform was used to detect more than 1700 mutations that correspond to 250 autosomal recessive diseases by SNPs array technique.
**Results:** The following table depicts the comparative results (prevalence) regarding to those reported by Recombine.

| Diseases | Female-Donors | Male-Donors | Reference (Recombine) |
|---|---|---|---|
| Cystic Fibrosis | 1:24 | 1:29 | 1:48 |
| No syndromic Hearing Loss and Deafness: GJB2 Related | 1:30 | 1:15 | 1:25 |
| Biotinidase Deficiency | 1:35 | 1:29 | 1:123 |
| Spinal Muscular Atrophy: SMN1 Linked | 1:27 | 0 | 1:55 |
| Fumarase Deficiency | 0 | 1:29 | 1:500 |
| 21HydroxylaseDeficient | | | |
| Nonclassical Congenital Adrenal Hyperplasia | 1:13 | 0 | 1:4 |
| Familial Mediterranean Fever | 1:51 | 1:29 | 1:4/1:80 |
| Pseudocholinesterase Deficiency | 1:51 | 1:15 | 1:33 |

**Conclusion:** The prevalence values were higher in comparison to those reported by the reference. It is clear that the screening of genetic diseases for oocyte and sperm donors is part of the necessary studies to reduce the risk of children born from this kind of treatment.

## P-42. Post-select spermatic DNA fragmentation index and its relation to embryonic parameters and clinical results

**A. Zevallos[1], R. Pella[1], C. Arévalo[1], S. Romero[1]**

**Objective:** Currently, the determination of sperm DNA fragmentation is frequently used for the evaluation of male infertility. There are numerous techniques for evaluating sperm DNA fragmentation, but the clinical implications on the development and maintenance of pregnancy remain heterogeneous. The aim of the present study was to determine the relationship between sperm DNA fragmentation index (DFI) of post-selection samples measured by the SCD (Sperm Chromatin Dispersion) assay and the rates of fertilization, blastulation, embryonic quality and clinical pregnancy in 71 couples treated by means of ICSI with ovodonation.
**Methods:** The study grouped non-asthenozoospermic samples that were used in treatment cycles and whose evaluation of post-selection sperm DNA fragmentation was not greater at 3 months. Four intervals were formed according to their DFI (≤13%; 14% - ≤26%; 27% - ≤39% and >39%).
**Results:** The rates of fertilization, blastulation and top-quality embryos on the fifth day did not show significant differences for fragmentation intervals or DFI. However, a statistically significant change ($P < 0.05$) was found for the pregnancy rate (60%, 31.25%, 0% and 0% respectively).
**Conclusion:** It is concluded that the index of sperm DNA fragmentation does not play an important role in the initial part of the embryonic development, but in the pregnancy rate. In addition, the DFI of a selected sample >13% should be considered for a treatment that allows a better selection of spermatozoa with non-fragmented DNA and thus increase the probability of pregnancies and births.

## P-43. Effects of the follicular fluid of women with endometriosis on in vitro maturation of mouse oocytes

**P. Berrío[1], I. Zorrilla[1], R. Pella[1], S. Romero[1]**

**Objective:** The objective of this study is to evaluate the effect of follicular fluid of women with endometriosis on in vitro maturation (IVM) of mouse oocytes.
**Methods:** Follicular fluids were obtained during follicular aspiration [1 patient with endometriosis (EFF) + 2 young oocyte donors (CFF)]. Forty F1 female mice (C57xBalb-c), aged 23-days, were primed with PMSG in order to induce follicular growth and allow the collection of immature oocytes at the germinal vesicle stage. After collection and selection, mouse cumulus-oocytes complexes (COCs) were divided into 7 groups and subjected to IVM: G0 (Control), G1 (20% CFF+0.3% BSA), G2 (10% CFF+0.3% BSA), G3 (5% CFF+0.3% BSA), G4 (20% EFF+0.3% BSA), G5 (10% EFF+0.3% BSA), G6 (5% EFF+0.3% BSA)
**Results:** When comparing maturation rate of G1 (94%±6%); G2 (95.7%±3.8%) and G3 (95.7%±7.5%) with the control group (82%±9.7%) there were not significantly differences ($P$=0.1610, $P$=0.1302, $P$=0.0740, respectively). Thus, follicular fluids of women without endometriosis does not affect mouse oocyte maturation. The maturation rate of G4 (50.2%±25.0%) was significantly lower than the control group (82%±9.7%), $P$=0.0169. Besides, G5 (57.8%±18.1%), was lower than the control group, however not significantly, $P$=0.0526.
**Conclusion:** In conclusion, this model of IVM assay allows us to evidence the negative effect of the follicular fluid of women with endometriosis on the mouse oocyte in vitro maturation system. Our findings suggest that follicular fluid of patients with endometriosis negatively affects the maturation (and perhaps the quality) of oocytes.

## P-44. Comparison of pregnancy rates in patients receiving oocytes from and egg-donation program and inseminated with ICSI or physiological ICSI

**M.A. Perez Padilla[1], F. Escudero[2], Y. Perez Solf[2], M. Garcia[2], P. Orihuela[2], R. Pella[1]**

**Objective:** The aim of the study was to evaluate the impact of the Hyaluronic-acid-sperm-selection in the pregnancy rate of an egg-donation program.
**Methods:** This retrospective study was performed in 187 cycles of the egg-donation program in the Center of Fertility and Assisted Reproduction (CEFRA). Two groups were considered: Conventional ICSI group (n=153) and Hyaluronic acid sperm-selection group (physiological ICSI or PICSI group, n=34). Fresh husband sperm specimens were used in both groups. After sperm selection, donated oocytes were injected. Oocytes with 2 pronuclei were considered as fertilized and were cultured in 50-ul drops of Global Total Medium (Life Global, USA) with oil overlay (Lite oil, Life global, USA) until the day of transfer (Day 5). The following parameters were assessed for both groups: sperm volume, concentration and motility; patient age, fertilization rate, and biochemical and pregnancy rate.
**Results:** Sperm selection with hyaluronic-acid in comparison to conventional ICSI does not improve biochemical pregnancy (80.4% and 76.5%, respectively), clinical pregnancy (74.5% and 70.58%, respectively) or blastulation rate (43.06 % and 44.62% respectively) in an egg-donation program.
**Conclusion:** In conclusion, hyaluronic acid sperm-selection does not improve the outcome, when using normal sperm samples and donor eggs.

## P-45. Influence of laser assisted hatching at day fourth of in vitro embryo development

**P. Villanueva[1,2], L. Noriega-Hoces[1,2], F. Vizcarra [1], G. Llerena[1,2], J. Portella[1,2], A. Villalobos[2], L. Guzmán[1,2]**

**Objective:** Laser assisted hatching (LAH) is been used to make a hole in the zona pellucid to help the extrusion of the blastocyst on day 5/6. However, it is still not known the effect of the laser on the embryo development. Objective: To determine if LAH performed on day four is affecting blastocyst formation.
**Methods:** For this retrospective study 2058 in vitro fertilization cycles were included. These cycles generated 11 486 blastocyst which were categorized following the SART morphological criteria as good, fair, and poor quality blastocyst. All remaining embryos were categorized as arrested embryos.
After embryo fertilization evaluation, embryos were cultured without interruption until day 4. AH was performed with laser (Lykos; Hamilton Thorne, Beverly, ME, USA). Then embryos were placed to continue their development until blastocyst formation. All statistical analysis were conducted with Stata v.14,
**Results:** Embryo categorized as: good quality embryo showed evidence of statistical association with age. In women under 35; 35-37; 38-40 41-42 and older than 43 and treated with laser AH, the good quality embryos was 28%; 25%; 22%; 21%; and 15% respectively. These results were statistically higher when they were compared to similar age match groups without intervention (23%; 17%; 15%; 10% and 7%).

**Conclusion:** LAH in the day four could be useful to assist embryo formation. Older women resulted beneficiated with laser intervention in terms of good blastocyst development. Further studies should be conducted to improve our knowledge in this field.

## P-46. Influence of repeated cycles of donor stimulation in the survival of vitrified oocytes

**J.A. Sánchez[1,2], E. Criado[1,2]**

[1]*FIV Marbella – Ovobank, Spain.*
[2]*Ovobank, Spain.*

**Objective:** Assess whether repeated stimulations in donors affects the quality of oocytes obtained in the follicular puncture, by oocyte survival after devitrification.
**Methods:** A total of 115 stimulations were analyzed, from 31 different donors submitted to more than 4 cycles of controlled ovarian stimulation. The maximum number of donations in a donor is 8, therefore 8 groups are created, each corresponding to the number of stimulations performed.
**Results:** The percentage of survival among the total devitrified oocytes is studied, no differences in the groups of 1 to 5 stimulations are observed, however they are observed in the group of 6 stimulations in the same donor. Similar results are obtained by calculating the average of surviving oocytes per follicular puncture. A lower total FSH consumption was observed between the groups of 2 and 3 stimulations, compared to the rest.
**Conclusion:** With the obtained results in this investigation we can conclude that the oocyte survival rate is not affected during the first 5 stimulations to which a donor is submitted. From the fifth stimulation, the oocyte survival shows a variation, due to the scarce data that we have from this one onwards. The difference in total FSH consumption in the groups of 2 and 3 stimulations is due to a change in the applied stimulation protocol in some of the donors. These studies would need to be completed to assess what incidence repeat cycles of stimulation in donors may have on oocyte quality.

## P-47. Application of software of egg bank management

**J.A. Sánchez[1,2], Enrique Criado[1,2]**

[1]*FIV Marbella, Spain.*
[2]*Ovobank, Spain.*

**Objective:** Check the improvement in the quality of the services offered with the OVOTRACKER application, compared to other databases previously used, with special emphasis on request-proposal-lot traceability. This improvement should be observed both in OVOBANK and in the collaborating centre that uses the service.
**Methods:** Use of the computer application by FIVMARBELLA, OVOBANK and collaborating centres' teams.
**Results:** Using OVOTRACKER significantly reduces errors generated in creating reports and documents required for the bank's management, because once the clinic introduces the request, all other necessary documentation is generated from such entered data, avoiding having to repeatedly write the same codes.
**Conclusion:**
1-OVOTRACKER is a development tool of its own, developed by all the departments of the clinic, which allows a better adjustment and flexibility of its benefits.
2- OVOTRACKER allows time saving, speeding up all tasks as it's an on-line platform.
3-OVOTRACKER offers traceability from the first moment we receive a request.
4-All necessary documents are generated automatically by the application, as they are not being created by OVOBANK personnel, human errors are minimized.
5-Finally, thanks to OVOTRACKER we can reduce the time spent in the management of the bank, enabling a double control in each of the steps performed, reducing human errors significantly.

## P-48. Transport and viability of cryopreserved oocytes

**C. González[1,2], E. Criado[1,2]**

[1]*FIV Marbella, Spain.*
[2]*Ovobank, Spain.*

**Objective:** Demonstrate that the conditions under which donated, vitrified oocytes are transported by air do not influence in the survival of the oocytes; neither the nitrogen vapours nor the changes in atmospheric pressure interfere with the survival and/or fertilization rates of the devitrified oocytes.
**Method:** Cryotops with vitrified oocytes were introduced into a Dry-Shipper container. This container is equipped with a probe that recorded the temperature throughout the trip, generating a graph to verify that the temperature was correct. The container was sent by air via "Lab to Lab" mode (without X-ray) to any destination and returned in the same way.
**Results:** 98 MII survived and 76 MII fertilized correctly. The data was compared to oocytes devitrified by the embryologists of our centre to those that had not been subjected to any type of transport. There were no differences in survival and fertilization rates between oocytes that had been transported by air and those that had not been transported at all.
**Conclusion:** The results obtained indicate that survival and fertilization are not lower when we devitrify oocytes that have been transported by air with the Lab to Lab system. Air transport and conditions do not seem to affect the survival and potential of vitrified donor oocytes.

## P-49. Investigation of the influence of body mass index in cycles of egg donation

**R. Sánchez[1,2], B. Martínez[1,2], E. Criado[1,2]**

[1]*FIV Marbella, Spain.*
[2]*Ovobank, Spain.*

**Objectives:** The overall objective is to assess whether there is a given BMI range, for which the rate of recovered Metaphase II (MII) eggs is increased. If it exists, the specific objective will be to determine the range of BMI for which the rate of recovered MII increases. The impact of BMI on the dose used will be studied.

**Method:** This is a retrospective investigation with a sample of 202 donation cycles, performed at FIV Marbella clinic. A statistical analysis has been carried out comparing the average of recovered MII's according to the established BMI ranges, following the guidelines of the World Health Organization.
**Results:** The results obtained show that when the BMI is <18.5 the average recovered MII in the different cycles is 20. With a BMI between 18.5 and 24.9 the average is decreased in one MII. Finally, with a BMI >25, a clear decrease of recovered MII is observed with an average of 16. It is observed that the existing correlation between BMI and dose used in the stimulation is negativized as BMI increases.
**Conclusion:** After analysing the obtained results, it can be observed that when the BMI is higher than 24.9 the effectiveness of the donation cycles is diminished as less MII are recovered. Highlighting that the lower the BMI, higher doses have been used not obtaining better results. Both results show that with a BMI between 18.5 and 24.9, the donation cycles obtained a higher success rate, taking into account recovered MII and dose used.

## P-50. Results FET D + 6 in single medium after 6 days vs single medium after 5 days of cultivation

**B. López[1,2], E. Criado[1,2]**

[1]*FIV Marbella, Spain.*
[2]*Ovobank, Spain.*

**Objective:** Assess if there is a difference between pregnancy rates (beta HCG) in day 6 cryotransfer cycles (FET D + 6), when the embryos have been cultured with a single medium for 6 days vs. 5 days of culture.
**Methods:** Good quality D + 6 Blastocysts from egg donation were cultivated in the FIV Marbella laboratory in a single medium (1-step Origio), and later vitrified. For the first group, the single culture medium prepared the day before microinjection was not replaced until the end of the culture (D + 6), it was used for 6 days; And in the second group the single culture medium also prepared the previous day, was replaced on D + 1, using for 5 days.
**Results:** Between January and December 2016 FET D+6 (n=21) were performed with embryos from the first group and FET D+6 (n=11) with embryos from the second group with significantly higher pregnancy rates for the second group versus first group. (38 vs. 54.5%).
**Conclusion:** We observed a significant difference in pregnancy rates between renewing the medium in D + 1 (group 2) and not doing so (group 1). The embryonic development could be compromised by the metabolism and the secretion of components to the culture medium after 6 days, being able to condition the quality of the vitrified blastocysts, which are subsequently transferred, interfering negatively in the achievement of a gestation. The preliminary studies of the data indicates that refreshing the medium could improve pregnancy rates in cryotransfer cycles in D + 6, but more studies will be needed.

## P-51. Comparison of 2 methods of insemination of oocytes (swim-out)

**C. González[1,2], E. Criado[1,2]**

[1]*FIV Marbella, Spain.*
[2]*Ovobank, Spain.*

**Objective:** To compare if there are differences in the rates of fertilization, blastulation and positive B-HCG using spermatozoa treated with density gradient against untreated sperm (Swim-out), directly from the ejaculate.
**Method:** The study was performed in 24 patients receiving donor oocytes and fresh semen samples from normozoospermic partners in the Fiv Marbella clinic.
We separated sample volume in a 5mL Falcon tube. The remainder of the sample was prepared using density gradients. Fifty percent of the oocytes from each donor were microinjected with treated semen and the other 50% with swim-out semen.
After 18 hours post-insemination the correct fertilization of oocytes (2 pronuclei and 2 polar bodies) was evaluated. The zygotes were cultured until day 5/6 in single medium (1-STEP, Source) being evaluated for transfer and/or vitrification.
**Results:** 97 MII were microinjected with treated semen and 94 MII with Swim-out semen. 10 embryo transfers from treated semen and 9 swim-out semen transfers were performed. The rate of fertilization, blastulation and positive B-hcg per transfer with treated semen were 77.3%, 68%, 70% and with swim-out semen 79.7%, 54.6%, 44.4% respectively.
**Conclusion:** According to our results, treating semen samples with gradients seems advisable to optimize the results in IVF-ICSI treatments, however we must continue to increase the number of treatments in order to draw meaningful conclusions.

## P-52. Percentage of aneuploidies embryos in egg donation treatments

**C. González[1,2], E. Criado[1,2]**

[1]*FIV Marbella, Spain.*
[2]*Ovobank, Spain.*

**Objective:** Evaluate the percentage of aneuploid embryos in PGS treatments with donor oocytes in our center, Fiv Marbella.
**Method:** We analyzed 48 egg donation treatments where screened for aneuploides was performed using karyolite or genomic hybridization array compared in the resulting embryos between 2015, 2016 and 2017. We calculated the percentage of aneuploid embryos according to the embryos analyzed by PGS.
**Results:** Of 279 embryos to which pre-implantation genetic diagnosis was performed, 117 were euploid, with 162 of them carrying some or several aneuploidies.
41.9% of the embryos analysed were suitable for transfer and/or freezing.
Studies performed in patients younger than 35 years of age show an euploid embryo rate of 40.84%.
**Conclusion:** According to our results, the rate of euploid embryos in egg donation treatments corresponds to the expected rate of euploid embryos in patients younger than 35 years of age, according to the studies performed by the genetic analysis laboratory where we perform our PGDs. Therefore, performing PGD on embryos from donor oocytes would be a way of ensuring a more effective transfer with a higher probability of evolutionary pregnancy. However, we must continue to expand this study by increasing the number of cases to reach meaningful conclusions.

## P-53. Success rates in egg donation treatments where the triple spermatic selection has been applied

**B. Renilla[1,2], E. Criado[1,2]**

[1]*FIV Marbella, Spain.*
[2]*Ovobank, Spain.*

**Objectives:** The purpose of this study is to demonstrate if there is a difference in the fertilization, blastulation and B-HCG positive rates, using the triple sperm selection (Gradients+MACs+PICSI), compared to treatments where conventional techniques have been applied.
**Methods:** The study was performed using donor oocyte recipients and their partner's sperm in fresh, applying the triple selection only in those samples that allowed the execution of the different laboratory techniques. In order to perform the triple sperm selection, we submit the sperm sample to a 100% SupraSperm density gradient (SAGE), the sperms obtained are passed through the MACs (MACs, Miltenyi Biotec) and lastly, the sperms retrieved are selected by PICSI. The average number of eggs used for each egg-donation treatment is approximately 9 oocytes for each recipient. The microinjection of the fresh eggs is performed 40h after the trigger.
**Results:** As we can see from the results (Without triple selection (n=119)/With triple selection (n=34)), fertilization rates (79.0%/78.9%), blastulation (67.8%/61.8%) do not present significant differences whereas in positive B-HCG (66.2%/53.5%) they do present significant differences between the different treatments.
**Conclusion:** According to our study, there are significant differences in the rate of positive B-HCG, although we must emphasize that the difference of "n" between treatments can be conclusive when giving a more reliable results. We will continue to work to increase the "n" of treatments with triple selection and to demonstrate if the differences may actually be significant or not.

## P-54. Cultivation to blastocyst without opening incubators

**B. Renilla[1,2], E. Criado[1,2]**

[1]*FIV Marbella, Spain.*
[2]*Ovobank, Spain.*

**Objectives:** The aim of this study is to demonstrate that in long culture up to blastocyst, not evaluating the embryos on day 3 can influence increasing blastulation rates and positive B-HCG, compared to treatments in which the embryos are valued on day 3.
**Methods:** The study was based on fertility treatments data of FIV Marbella clinic: treatments with the couple's own eggs, donor eggs, and partner's semen or donor samples. The treatment of the sperm sample was performed using gradients, MACs and PICSI according to the treatment requirements. The microinjection of the oocytes was performed 40hrs after the trigger and 1hr after the devitrification if the oocytes used had previously been cryopreserved. The prolonged culture of the embryos was in a single medium, with the embryos being evaluated on D+1, on D+3 and later on D+5. In the same way a long culture protocol was implemented by evaluating the embryos on D+1 and D+5.
**Results:** We see that there are no significant differences in fertilization rates, however we do see a significant increase in blastulation rates (55.0%/67.9%) and positive B-HCG (54.8%/60.7%), in those treatments in which the embryos had not been valued on D+3.
**Conclusions:** We can conclude that by not altering the culture conditions from the evaluation of fertilization until the day of the transfer on D+5, blastulation rates and positive B-HCG can be increased.


Chaquiriand V. 1
1 Centro de Esterilidad Montevideo, Uruguay

P-07. Randomized comparison of two commercial culture media (Cook and Vitrolife) for embryo culture after IMSI

Petersen C.G. 1 2
Mauri A.L. 1 2
Vagnini L.D. 2
Renzi A. 2
Petersen B. 2
Matilla M. 1
Comar V. 1
Ricci J. 1
Dieamant F. 1
Oliveira J.B.A. 1 2
Baruffi R.L.R. 1 2
Franco Jr J. G. 1 2
1 Center for Human Reproduction Prof. Franco Jr, Ribeirao Preto, Brazil
2 Paulista Center for Diagnosis Research and Training, Ribeirao Preto, Brazil

P-08. Early serum progesterone and prolactin analysis at day 9 of follicular uptake as a predictor of success in fresh ICSI cycles

Pérez P.A.S. 1
Ceschin A.P. 1
1 Feliccità Instituto de Fertilidade, Curitiba, Brazil

P-09. Healthy birth following intracytoplasmic sperm injection from ejaculated sperm after bilateral testicular sperm extraction failure

Macedo J.F. de 1
Macedo G. C. de 2
Macedo G. C. de 2
Campos L.A. 1 3
Baltatu O.C. 3
1 Reproferty Reproductive Medicine Clinic, Sao Jose dos Campos, Brazil
2 Faculty of Medicine, Estácio de Sá University, Rio de Janeiro, Brazil
3 Center of Innovation, Technology and Education (CITE), Anhembi Morumbi University-Laureate International Universities, Sao Jose dos Campos, Brazil

P-10. Study of the main reasons for rejection

Casado M. 1 2
Martínez B. 1 2
Criado E. 1 2
1 FIV Marbella, Spain
2 Ovobank, Spain

P-11. Uninterrupted embryo culture. Development of blastocysts using a continuous culture system without medium renewal

Veiga M.F. 1
Rivadeneira A. 1
Giordana S. 1
Neuspiller F. 1
1 IVI Buenos Aires - Argentina

P-12. Importance of the diagnostic hysteroscopy in an in vitro fertilization program: experience in our center

Blum-Narváez M. 1
Puga-Torres T. 1
Blum-Pinto B. 1
Arce-Burgos I. 1
Espinoza-Constante B. 1
1 Centro Nacional de Reproducción Asistida INNAIFEST - Guayaquil - Ecuador

P-13. ART using washing semen in serodiscordant couples

Reig V. 1
Proença L.A. 1
Flach S.W. 1
Kvitko D.T. 1
Tagliani-Ribeiro A. 1
Okada L. 1
Azambuja R. 1
Petracco A. 1
Badalotti M. 1
1 Fertilitat-Centro de Medicina Reprodutiva. Porto Alegre, RS. Brasil

P-14. Effect of MACs on sperm DNA fragmentation and survival rate

Grela M.E. 1
Olivieri M.T. 2
Díaz-Acacio A. 2
Bronfenmajer S. 2
1 Universidad Simón Bolívar. Caracas, Venezuela
2 EMBRIOS. Hospital de Clínicas Caracas. Caracas, Venezuela

P-15. Management of a simple blastocyst qualification system in embryo selection for transfer

Puga-Torres T. 1
Blum-Rojas X. 1
Blum-Narváez M. 1
1 Centro Nacional de Reproducciónn Asistida INNAIFEST - Guayaquil - Ecuador

P-16. "Freeze-all" policy in poor diagnosis patients

Cicare J. 1
Hovanyecz P. 1
Perfumo P. 1
Domenech L. 1
Ventura V. 1
Paz M.V. 1
1 Sanatorio Los Arroyos, Grupo Gamma, Rosario, Argentina

P-17. Multinucleated embryo transfers and its impact on clinical outcomes

Hovanyecz P. 1
Cicaré J. 1
Domenech L. 1
Perfumo P. 1
Ventura V. 1
Paz M.V. 1
1 Sanatorio Los Arroyos. Grupo Gamma. Rosario, Argentina

P-18. Single versus group embryo cultures until day three of development

Frautschi C. 1
Agostino A. D' 1
Dematteis A. 1
Estofan G. 1
Estofan D. 1
Hernández M. 1
1 CIGOR. Centro Integral de Ginecología, Obstetricia y Reproducción. Córdoba, Argentina

P-19. Aneuploid screening results: first IVI Santiago report

Machuca D. 1
Andrés E. 1
Troncoso C. 1
Al-Asmar N. 1
Rubio C. 1
Calonge M. 1
1 IVI Santiago, Chile

P-20. Evaluation of seminal quality in patients with problems of fertility of the Human Reproduction Center of Lima (NACER)

Diaz Pinillos J.V. 1
Burga Davila L. 1
Human Reproduction Center of Lima (NACER) - Peru

P-21. General public accessing ART: interlinking between public and private institutions

Mas de Ayala J. 1
Barrera N. 1
Alciaturi J. 1
Bonelli C. 1
García R. 1
Cantú L. 1
1 Laboratorio de Reproducción Asistida, Centro de Esterilidad Montevideo, Montevideo, Uruguay

P-22. First reported of Double Ovarian Stimulation (DOS) in a crease cancer patient, an effective toll for fertility preservation

Raso D. 1
Lotti B. 1
Barattini J. 1
Marconi G. 1
Neuspiller F. 1
1 IVI Buenos Aires - Argentina

P-23. Endometrial Receptivity Analysis: a useful tool for personalized embryo transfer in patients with related implantation failure

Lotti B. 1
Raso D. 1
D'Atri F. 1
Fernández Peri N. 1
Neuspiller F. 1
1 IVI Buenos Aires - Argentina

P-24. Non-treated male accessory gland in section syndrome (MAGI-S) may impair semen quality

Avendaño C. 1 2 3 4
Ferrer D. 1 2
Ojeda J. 5
Gallardo G. 1 2
Juárez Villanueva A. 1 2
1 Gynesis, Córdoba, Argentina
2 Alive Sanatorio Privado, Córdoba, Argentina
3 LAC Trelew, Chubut ,Argentina
4 Dibac Trelew, Chubut, Argentina
5 Sanatorio Trelew, Chubut, Argentina

P-25. Routinely freezing al blastocyst and transfer in a non-gonadotrophin stimulated cycle as a strategy to increase pregnancy outcomes in IVF

Abdala A. 1
Nuñez M. 2
Gómez Passanante E. 1
Raffo F. 1
Provenzano S. 1
Singla J. 1
1 Unidad de Fertilización Asistida, División ginecología, Departamento de Tocoginecología, Hospital de Clínicas "José de San Martin", Universidad de Buenos Aires
2 Cátedra de Matemática, Facultad de Farmacia y Bioquímica, Universidad de Buenos Aires, Argentina

P-26. The endometrial from women with recurrent implantation failure exhibit abnormal expression of complement regulatory proteins. A promising immunologic marker of endometrial receptivity

Palomino A. 1
Aguirre C. 1
Argandoña F. 1
Sequeira K. 1
Devoto L. 1
1 Instituto de Investigaciones Materno Infantil, Unidad de Medicina Reproductiva, Universidad de Chile, Santiago, Chile

P-27. A preliminary retrospective study on sperm banking. The Uruguayan experience

Ordoqui R. 1
Barrera N. 1
Montes J.M. 1
Canepa M. 1
Bonelli C. 1
Surka C. 1
Torrens A. 1
Cantú L. 1
Du Plessis S.S. 2
1 Fertilab Laboratorio de Análisis Clínicos, Laboratorio de Andrología, Montevideo, Uruguay
2 Medical Physiology, Faculty of Medicine and Health Sciences, Stellenbosch University, Tygerberg, South Africa

P-28. Maternal age and aneuploidies by Next Generation Sequencing (NGS)

Villanueva P. 1 2
Noriega-Hoces L. 1 2
Vizcarra F. 1
Portella J. 1 2
Lópezi R. 3
Villalobos A. 2
Guzmáni L. 1 2 3
1 PRANOR. Grupo de Reproducción Asistida. San Isidro. Lima-Peru
2 Clínica Concebir. San Isidro. Lima-Peru
3 Reprogenetics Latinoamérica. Lima-Peru

P-29. Oocyte vitrification in a donation program

Mata A. 1
Pérez M. 1
Maino C. 1
Heredia J. 1
Pérez Alzaa J. 1
1 Fundación Fecundart, Córdoba, Argentina

P-30. Mosaic embryo transfer after oocyte in vitro maturation in combination with non-invasive prenatal test (NIPT) - First report of a live birth

Inoue N. 1
Lopez R. 2
Delgado A. 3
Nuñez D. 1
Portella J. 1
Noriega-Hoces L. 1 3
Guzmán L. 1 2
1 PRANOR. Grupo de Reproducción Asistida. San Isidro. Lima-Peru
2 Reprogenetics Latinoamerica, Lima, Peru
3 Clínica Concebir. San Isidro. Lima-Peru

P-31. Sequential aneuploidies in human blastocyst and their relation with maternal age

Sánchez Usabiaga R.A. 1
Regeyra Edelman C. 1
Duran Montaño C. 1
Ramirez Rivera E.G. 1
Gonzalez Becerra E. 1
1 Médica Fértil - Mexico

P-32. Possible influence of the different shipment types on the viability of vitrified oocytes

López B. 1 2
Criado E. 1 2
1 FIV Marbella - Ovobank, Spain
2 Ovobank, Spain

P-33. Delayed blastocyst formation and its influence on frozen-trowed transfer. Day 5 vs Day 6

Paz M.V. 1
Cicaré J. 1
Hovanyecz P. 1
Domenech L. 1
Perfumo P. 1
Ventura V. 1
1 Sanatorio Los Arroyos, GrupoGamma, Rosario, Argentina

P-34. Gender differences in dealing with assisted reproduction treatment: psychological and social reaction in men and women

Lopes H. P. 1
Avelar C. 2
1 Pró Nascer - Reprodução Humana, Rio de Janeiro, RJ, Brazil
2 Pró-Criar Medicina Reprodutiva , Belo Horizonte , Brazil

P-35. Results of intrauterine insemination versus intracytoplasmic sperm injection in patients between 40 and 43 years

Lucini C. 1
Pasqualini A. 1
Aza Y. 1
Piscicelli C. 1
1 Instituto Médico Halitus, Buenos Aires, Argentina

P-36. Psychopathological status, previous infertility diagnosis and failed fertility treatments in members of infertile couples

Jurkowski L. 1
Balbi P. 1
Bossi N. 1
Granados C. 1
Polak de Fried E. 1
1 CER Medical Institute, affiliated to the Universidad de Buenos Aires, Argentina

P-37. Embryo transfer quality evaluation performed by clinicians and embryologists. Pregnancy rate impact

Notrica J. 1
Granados C. 1
Bossi N. 1
Polak de Fried E. 1
1 CER Medical Institute, affiliated to the Universidad de Buenos Aires, Argentina

P-38. Sperm DNA fragmentation (Tunel) in the evaluation of male infertility a new cut-off value

Alvarez Sedó C. 1
Bilinski M. 1
Lorenzi D. 1
Miguens M. 1
Borghi C. 1
Papier S. 1
1 Centro de Estudios en Genética y Reproducción (CEGYR), Buenos Aires, Argentina

P-39. Blastocyst mitochondrial DNA quantification and its association with female age, aneuploidy and implantation

Alvarez Sedó C. 1
Bilinski M. 1
Lorenzi D. 1
Fabbro M. 1
Nodar F. 1
Papier S. 1
1 Centro de Estudios en Genética y Reproducción (CEGYR), Buenos Aires, Argentina

P-40. Fetal fraction in maternal plasma could be affected by fetal aneuploidy and maternal weight experience in 1600 NIPT cases

Alvarez Sedó C. 1
Nicotra P. 1
Fiszbajn G. 1
Kopelman S. 1
Papier S. 1
Hamer J. 1
1 Centro de Estudios en Genética y Reproducción (CEGYR), Buenos Aires, Argentina

P-41. Genetic carrier screening in sperm and oocyte donors

Alvarez Sedó C. 1
Quinteiro Retamar A. 1
Borghi C. 1
De Carvalho P. 1
Nodar F. 1
Papier S. 1
1 Centro de Estudios en Genética y Reproducción (CEGYR), Buenos Aires, Argentina

P-42. Post-select spermatic DNA fragmentation index and its relation to embryonic parameters and clinical results

Zevallos A. 1
Pella R. 1
Arévalo C. 1
Romero S. 1
1 Centro de Fertilidad y Reproducción Asistida (CEFRA), Lima, Peru

P-43. Effects of the follicular fluid of women with endometriosis on in vitro maturation of mouse oocytes

Berrío P. 1
Zorrilla I. 1
Pella R. 1
Romero S. 1
1 Centro de Fertilidad y Reproducción Asistida CEFRA, Laboratorio de Embriología, Lima, Peru

P-44. Comparison of pregnancy rates in patients receiving oocytes from and egg-donation program and inseminated with ICSI or physiological ICSI

Perez Padilla M.A. 1
Escudero F. 2
Perez Solf Y. 2
Garcia M. 2
Orihuela P. 2
Pella R. 1
1 Centro de Fertilidad y Reproducción Asistida CEFRA, Laboratorio de Embriología, Lima, Peru
2 Centro de Fertilidad y Reproducción Asistida CEFRA, Unidad Clínica, Lima, Peru

P-45. Influence of laser assisted hatching at day fourth of in vitro embryo development

Villanueva P. 1 2
Noriega-Hoces L. 1 2
Vizcarra F. 1
Llerena G. 1 2
Portella J. 1 2
Villalobos A. 2
Guzmán L. 1 2
1 PRANOR. Grupo de Reproducción Asistida. San Isidro. Lima-Peru
2 Clínica Concebir. San Isidro. Lima-Peru

P-46. Influence of repeated cycles of donor stimulation in the survival of vitrified oocytes

Sánchez J.A. 1 2
Criado E. 1 2
1 FIV Marbella - Ovobank, Spain
2 Ovobank, Spain

P-47. Application of software of egg bank management

Sánchez J.A. 1 2
Criado Enrique 1 2
1 FIV Marbella, Spain
2 Ovobank, Spain

P-48. Transport and viability of cryopreserved oocytes

González C. 1 2
Criado E. 1 2
1 FIV Marbella, Spain
2 Ovobank, Spain

P-49. Investigation of the influence of body mass index in cycles of egg donation

Sánchez R. 1 2
Martínez B. 1 2
Criado E. 1 2
1 FIV Marbella, Spain
2 Ovobank, Spain

P-50. Results FET D + 6 in single medium after 6 days vs single medium after 5 days of cultivation

López B. 1 2
Criado E. 1 2
1 FIV Marbella, Spain
2 Ovobank, Spain

P-51. Comparison of 2 methods of insemination of oocytes (swim-out)

González C. 1 2
Criado E. 1 2
1 FIV Marbella, Spain
2 Ovobank, Spain

P-52. Percentage of aneuploidies embryos in egg donation treatments

González C. 1 2
Criado E. 1 2
1 FIV Marbella, Spain
2 Ovobank, Spain

P-53. Success rates in egg donation treatments where the triple spermatic selection has been applied

Renilla B. 1 2
Criado E. 1 2
1 FIV Marbella, Spain
2 Ovobank, Spain

P-54. Cultivation to blastocyst without opening incubators

Renilla B. 1 2
Criado E. 1 2
1 FIV Marbella, Spain
2 Ovobank, Spain

Claudio Chillik Award

Best Poster Presentation.

13º REDLARA General Congress – Buenos Aires – Argentina 2017

